# Supplementary material for: Late Cretaceous ammonoids show that drivers of diversification are regionally heterogeneous
Source: Nat Commun. 2024 Jun 27;15:5382. doi: 10.1038/s41467-024-49462-z (PMC11211348; doi:10.1038/s41467-024-49462-z)
Supplement: Supplementary file 1 — Supplementary Information [file 41467_2024_49462_MOESM1_ESM.pdf]

Supplementary information

Flannery-Sutherland et al. (2024). Late Cretaceous ammonoids show that drivers of diversification are regionally heterogeneous.

Note that the electronic supplement containing our code and data is separate to this file and available at: https://doi.org/10.6084/m9.figshare.25563633

Supplementary Figures 1–9. Occurrence counts, spatial extent, and subsampled diversity of Late Cretaceous ammonoids before and after spatial standardisation. Solid lines are for metrics before spatial standardisation while dotted lines are for metrics after spatial standardisation. Red lines are longitudinal range while green lines are subsampled diversity at a sampling quorum of 0.5. Source data is available in the electronic supplement accompanying the paper.

Supplementary Figures 10–18. Late Cretaceous ammonoid diversification rates from spatially standardised regional and global datasets. Solid lines are for metrics before spatial standardisation while dotted lines are for metrics after spatial standardisation. Each plot displays the mean and 95% highest posterior density for each rate estimate. Source data is available in the electronic supplement accompanying the paper.

Supplementary Figures 19–27. Late Cretaceous ammonoid exponential multivariate birth-death correlations from spatially standardised datasets. W refers to the shrinkage weight of the correlation where W > 0.5 is statistically significant. Div. = diversity; ichth. = ichthyolith; surf. – surface. Source data is available in the electronic supplement accompanying the paper.

Supplementary Figures 28–36. Late Cretaceous ammonoid linear multivariate birth-death correlations from spatially standardised datasets. W refers to the shrinkage weight of the correlation where W > 0.5 is statistically significant. Div. = diversity; ichth. = ichthyolith; surf. – surface. Source data is available in the electronic supplement accompanying the paper.

Supplementary Table 1. Correlations between Late Cretaceous ammonoid spatial extent and subsampled diversity. Pearson's and Spearman's correlation strength and statistical significance (p) are reported for each test.

Supplementary Table 2. Fits of PyRate preservation models to spatially standardised regional and global Late Cretaceous ammonoid occurrence datasets. Models fitted were the homogenous Poisson process, non-homogenous Poisson process, and the piece-wise constant through-time Poisson process.

Supplementary Table 3. Fits of exponential and linear birth-death correlation models to regional and global Late Cretaceous ammonoid diversification rates. The differences in the AIC scores (ΔAIC) generally show that one model does not outperform the other. Log-likelihoods (lnl) of each model were used to calculate log Bayes Factor support values in favour of the exponential model. The log Bayes Factors generally show that one model does not outperform the other.

Supplementary Tables 4–12. Multivariate birth-death correlations for regional and global Late Cretaceous ammonoid diversification rates at genus and species levels. Each table displays mean and 95% highest posterior density of each correlation value, where $W > 0.5$ is taken as statistically significant.

Supplementary Table 13. Suborder predictors of regional and global Late Cretaceous ammonoid extinction risk. Each table displays the combined statistical significance of all predictors (I), where $I > 0.5$ is significant, and the individual effect strengths of each taxonomic predictor. Note that this table is split over two pages.

Supplementary Table 14. Superfamily predictors of regional and global Late Cretaceous ammonoid extinction risk. Each table displays the combined statistical significance of all predictors (I), where $I > 0.5$ is significant, and the individual effect strengths of each taxonomic predictor. Note that this table is split over two pages.

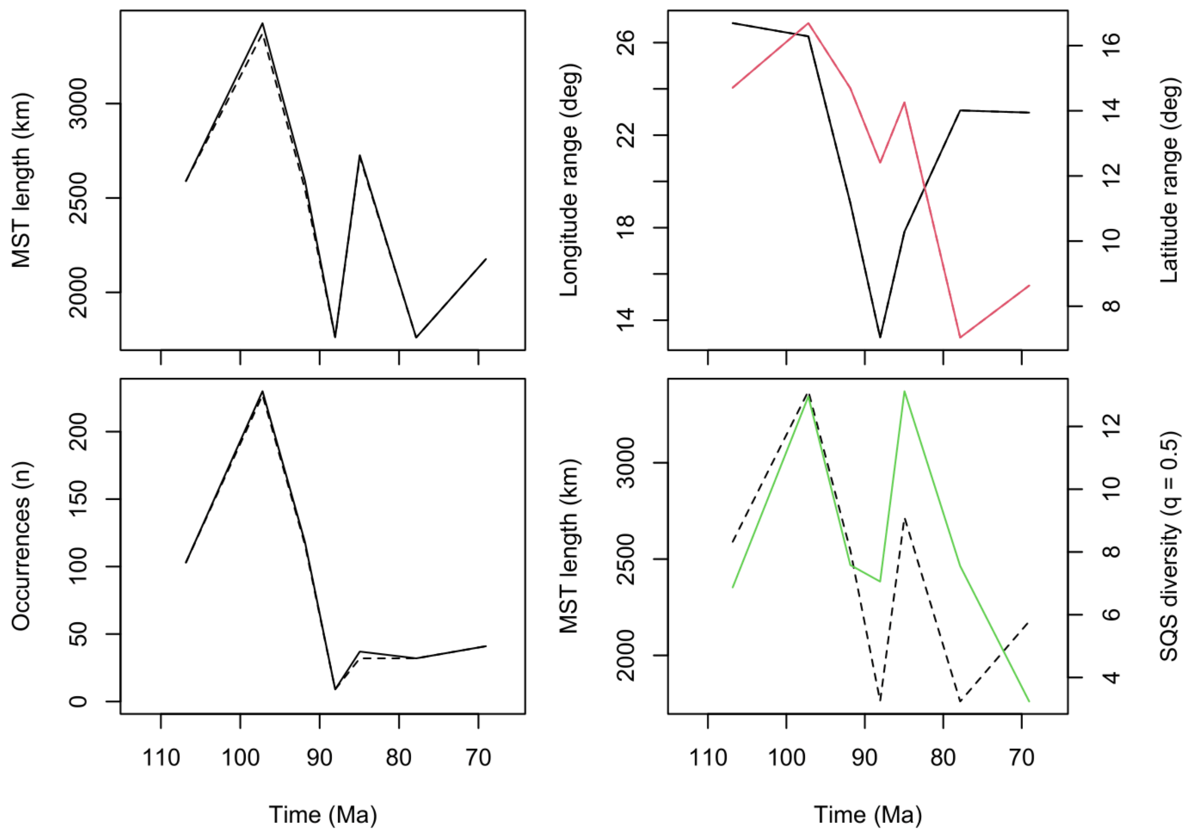

Supplementary Fig. 1. Spatial standardisation of Antarctic ammonoid occurrences. Solid black lines are for metrics before spatial standardisation while dotted black lines are for metrics after spatial standardisation. Red lines are longitudinal range while green lines are subsampled diversity at a sampling quorum of 0.5. Source data is available in the electronic supplement accompanying the paper.

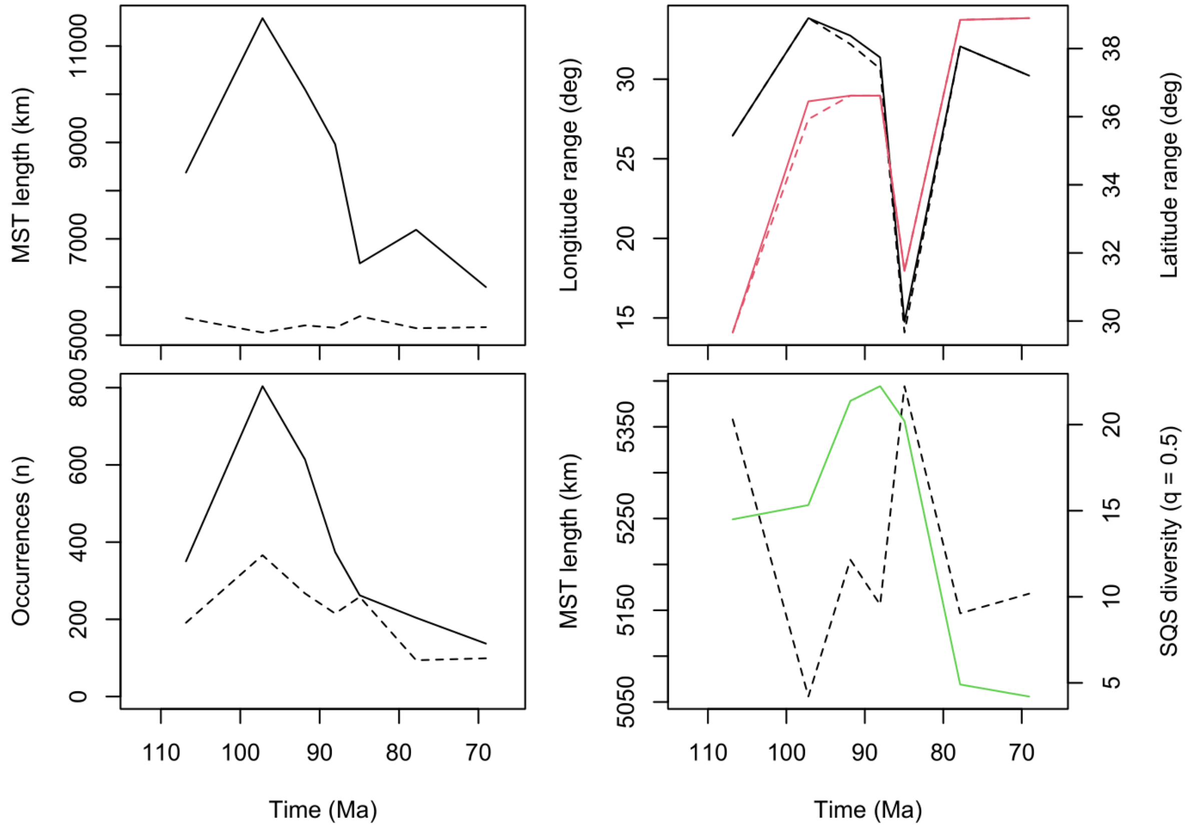

Supplementary Fig. 2. Spatial standardisation of East Pacific ammonoid occurrences. Solid black lines are for metrics before spatial standardisation while dotted black lines are for metrics after spatial standardisation. Red lines are longitudinal range while green lines are subsampled diversity at a sampling quorum of 0.5. Source data is available in the electronic supplement accompanying the paper.

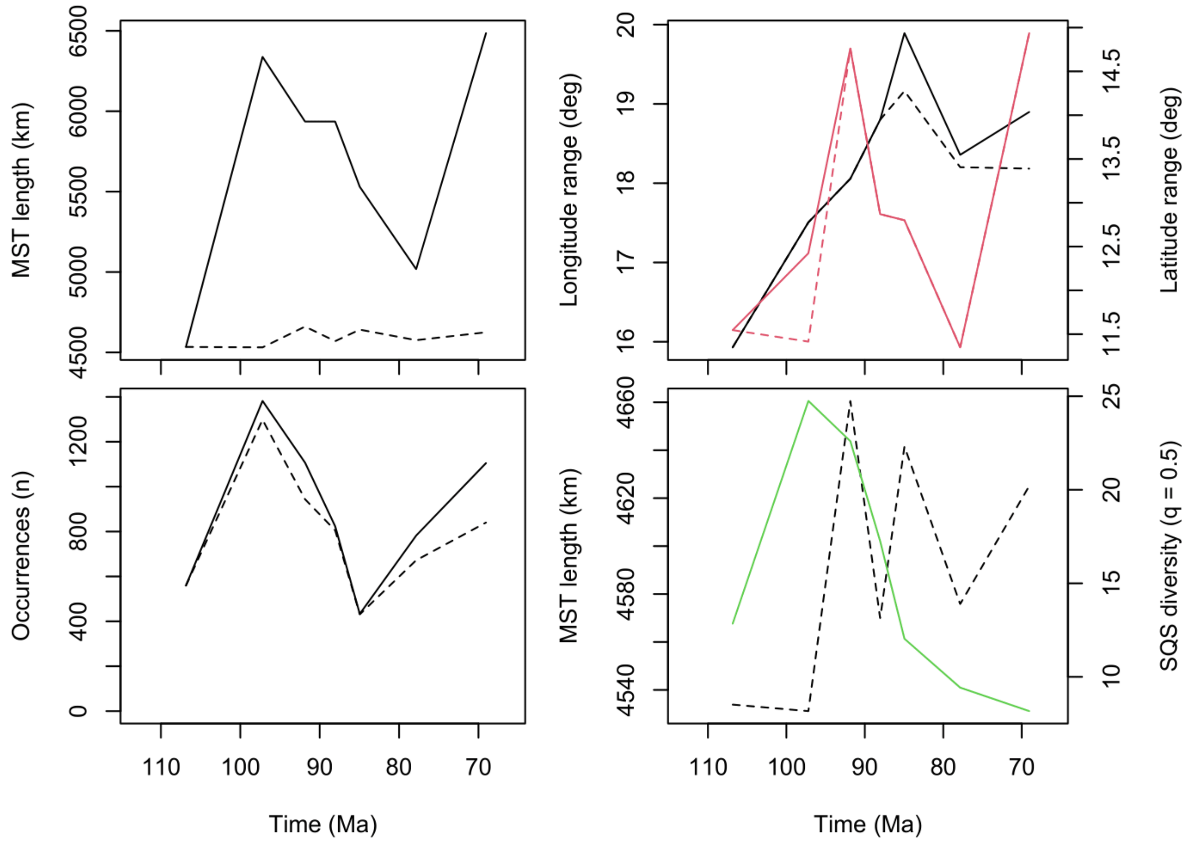

Supplementary Fig. 3. Spatial standardisation of Atlantic and Gulf ammonoid occurrences. Solid black lines are for metrics before spatial standardisation while dotted black lines are for metrics after spatial standardisation. Red lines are longitudinal range while green lines are subsampled diversity at a sampling quorum of 0.5. Source data is available in the electronic supplement accompanying the paper.

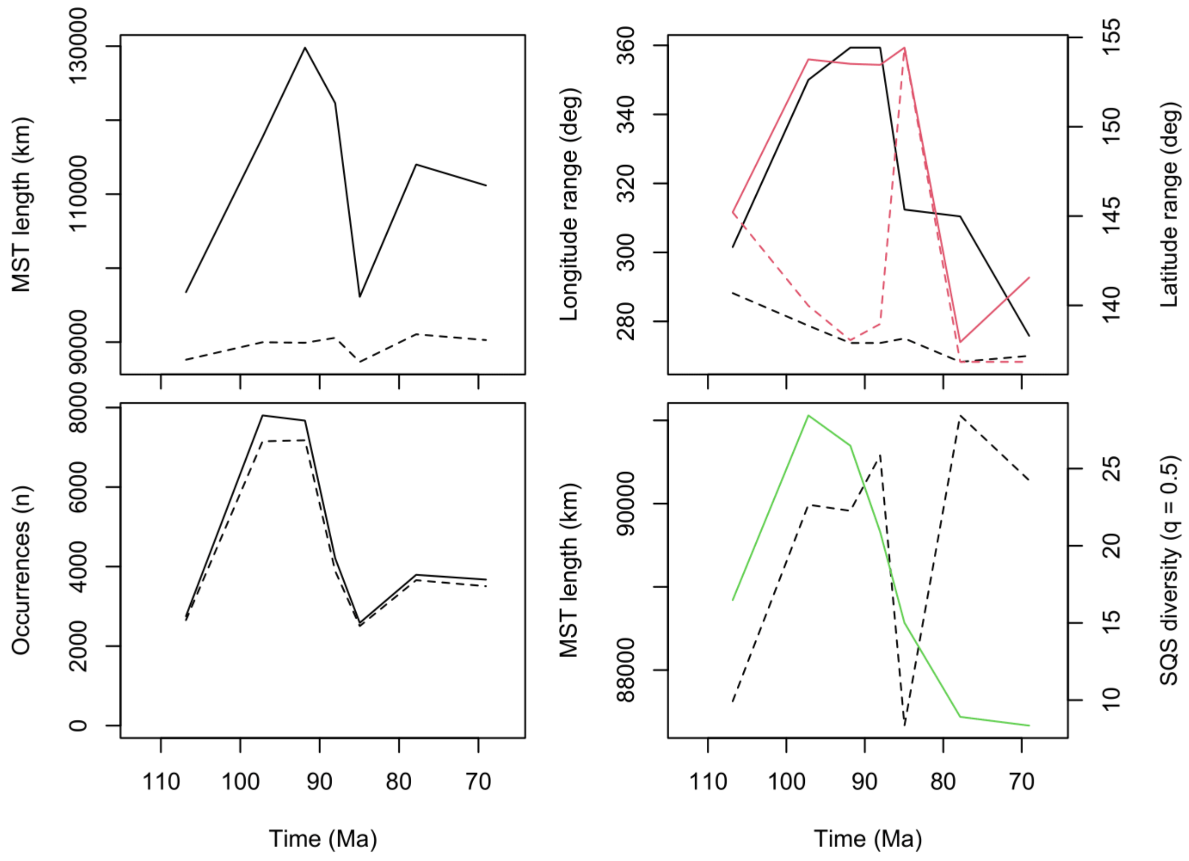

Supplementary Fig. 4. Spatial standardisation of global ammonoid occurrences. Solid black lines are for metrics before spatial standardisation while dotted black lines are for metrics after spatial standardisation. Red lines are longitudinal range while green lines are subsampled diversity at a sampling quorum of 0.5. Source data is available in the electronic supplement accompanying the paper.

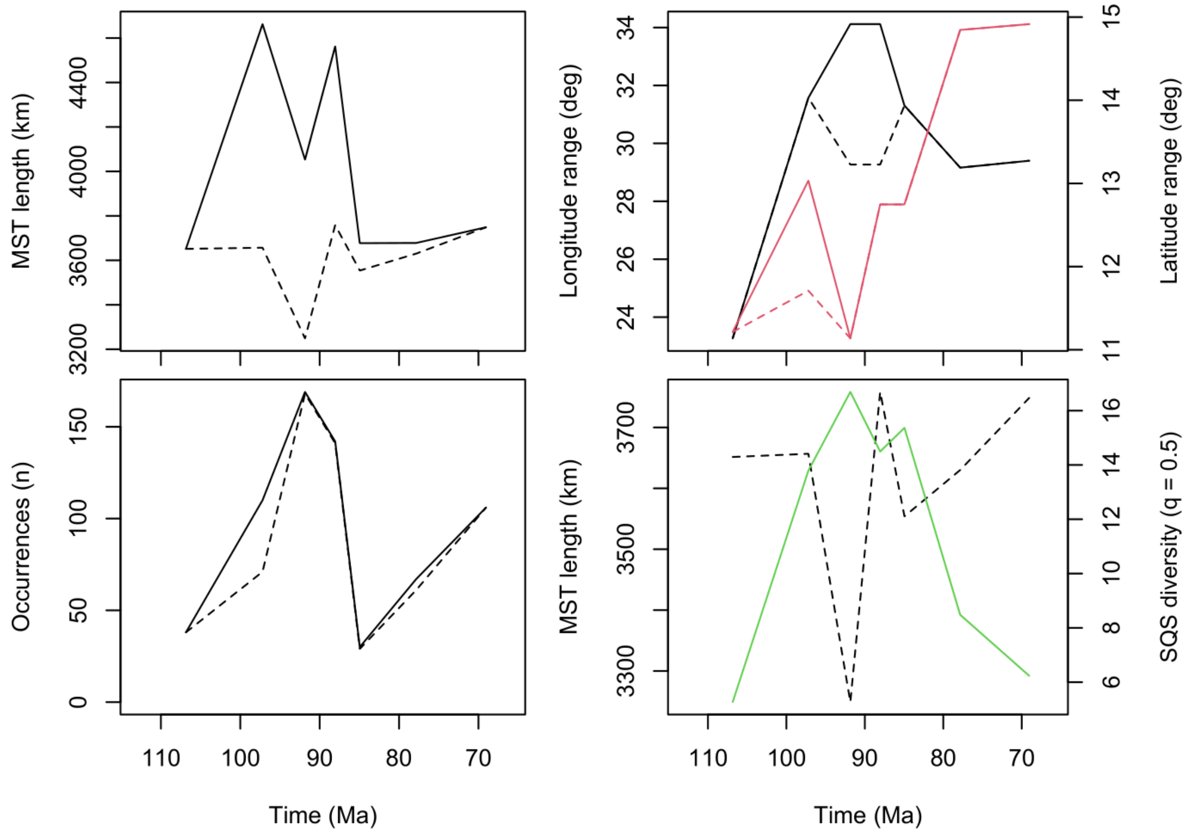

Supplementary Fig. 5. Spatial standardisation of South African ammonoid occurrences. Solid black lines are for metrics before spatial standardisation while dotted black lines are for metrics after spatial standardisation. Red lines are longitudinal range while green lines are subsampled diversity at a sampling quorum of 0.5. Source data is available in the electronic supplement accompanying the paper.

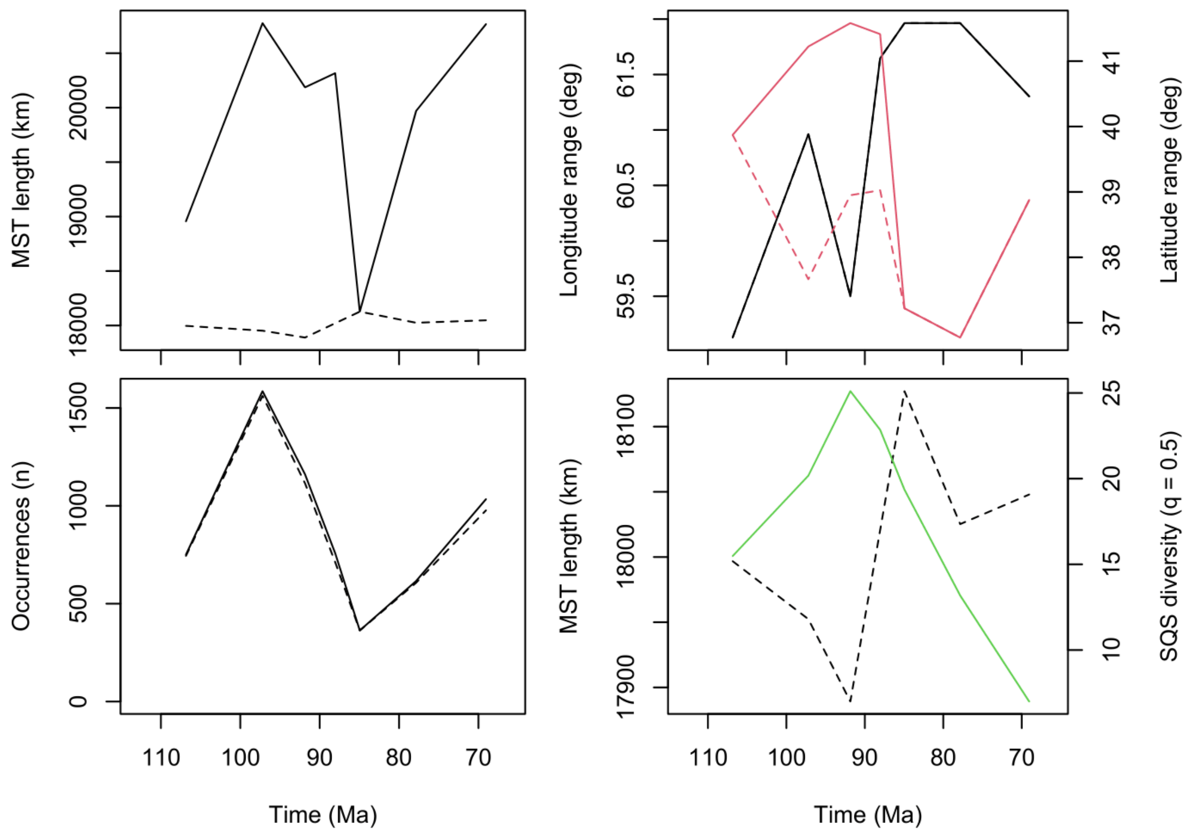

Supplementary Fig. 6. Spatial standardisation of Tethyan ammonoid occurrences. Solid black lines are for metrics before spatial standardisation while dotted black lines are for metrics after spatial standardisation. Red lines are longitudinal range while green lines are subsampled diversity at a sampling quorum of 0.5. Source data is available in the electronic supplement accompanying the paper.

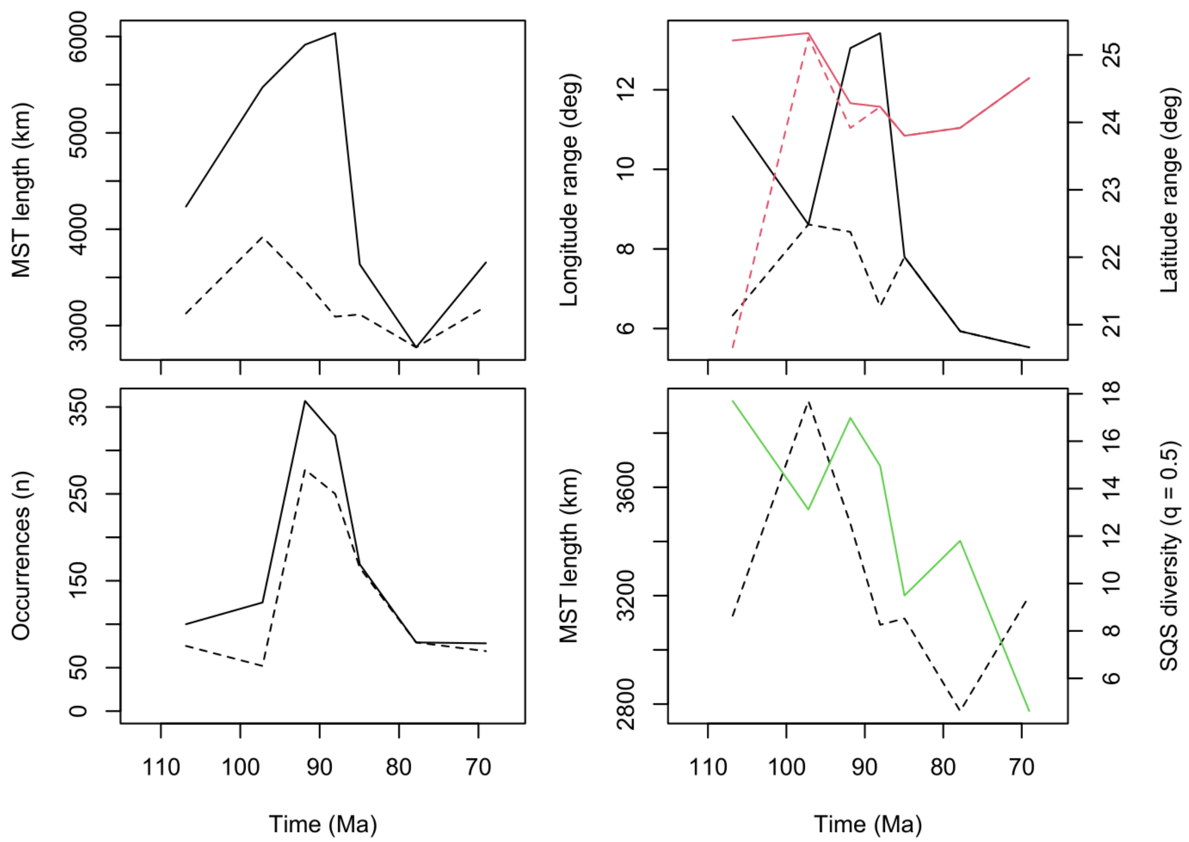

Supplementary Fig. 7. Spatial standardisation of West African ammonoid occurrences. Solid black lines are for metrics before spatial standardisation while dotted black lines are for metrics after spatial standardisation. Red lines are longitudinal range while green lines are subsampled diversity at a sampling quorum of 0.5. Source data is available in the electronic supplement accompanying the paper.

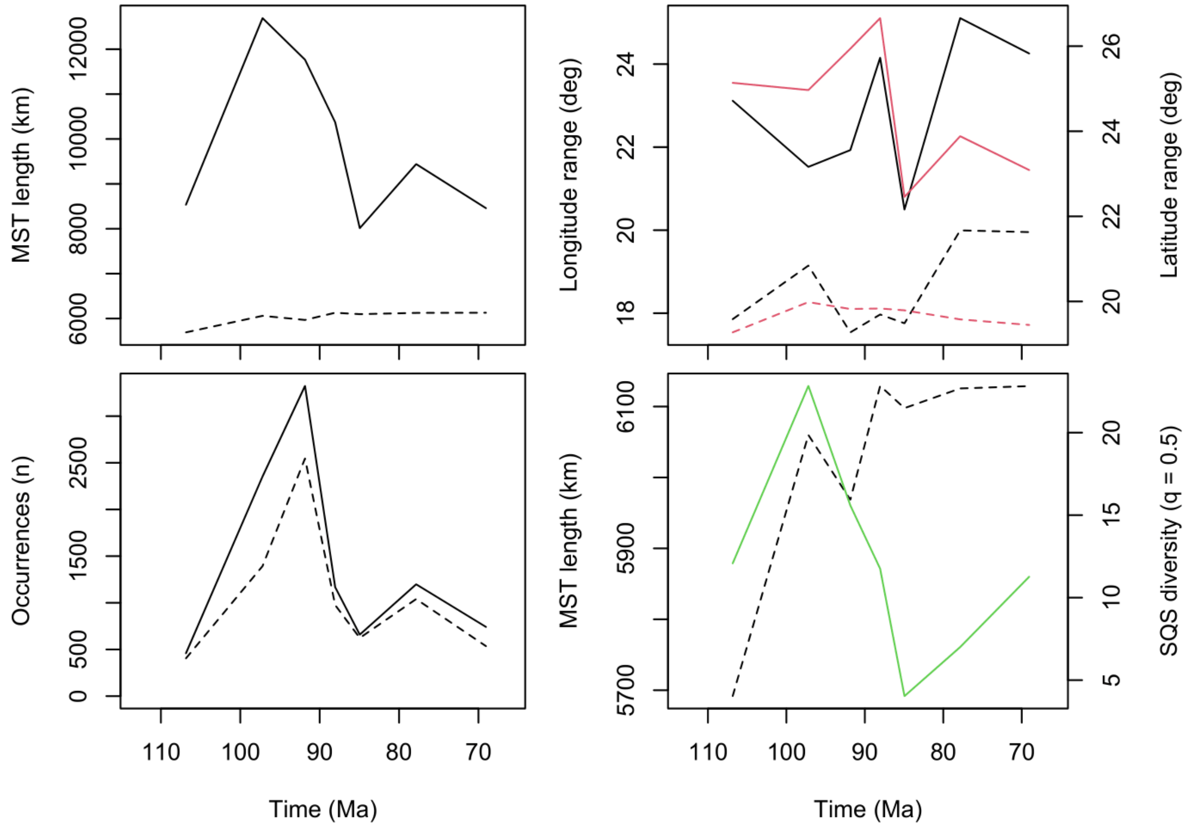

Supplementary Fig. 8. Spatial standardisation of Western Interior Seaway occurrences. Solid black lines are for metrics before spatial standardisation while dotted black lines are for metrics after spatial standardisation. Red lines are longitudinal range while green lines are subsampled diversity at a sampling quorum of 0.5. Source data is available in the electronic supplement accompanying the paper.

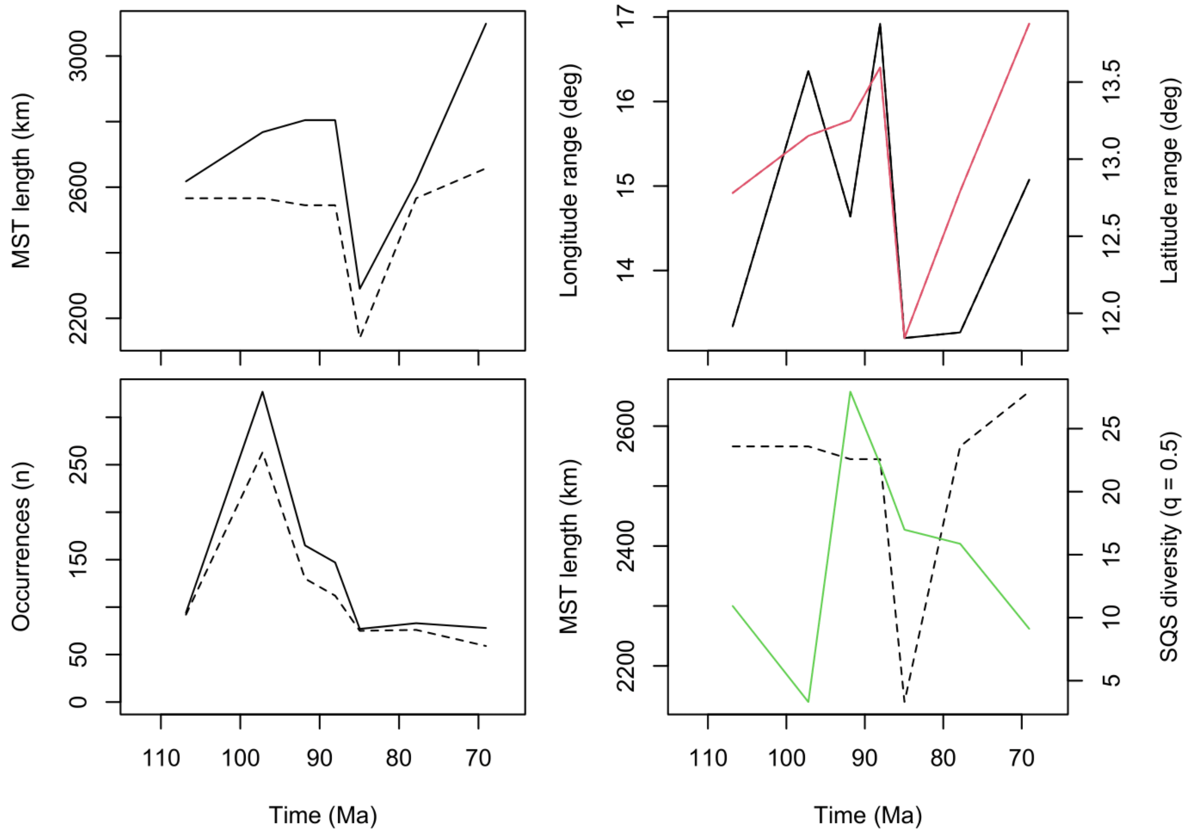

Supplementary Fig. 9. Spatial standardisation of West Pacific ammonoid occurrences. Solid black lines are for metrics before spatial standardisation while dotted black lines are for metrics after spatial standardisation. Red lines are longitudinal range while green lines are subsampled diversity at a sampling quorum of 0.5. Source data is available in the electronic supplement accompanying the paper.

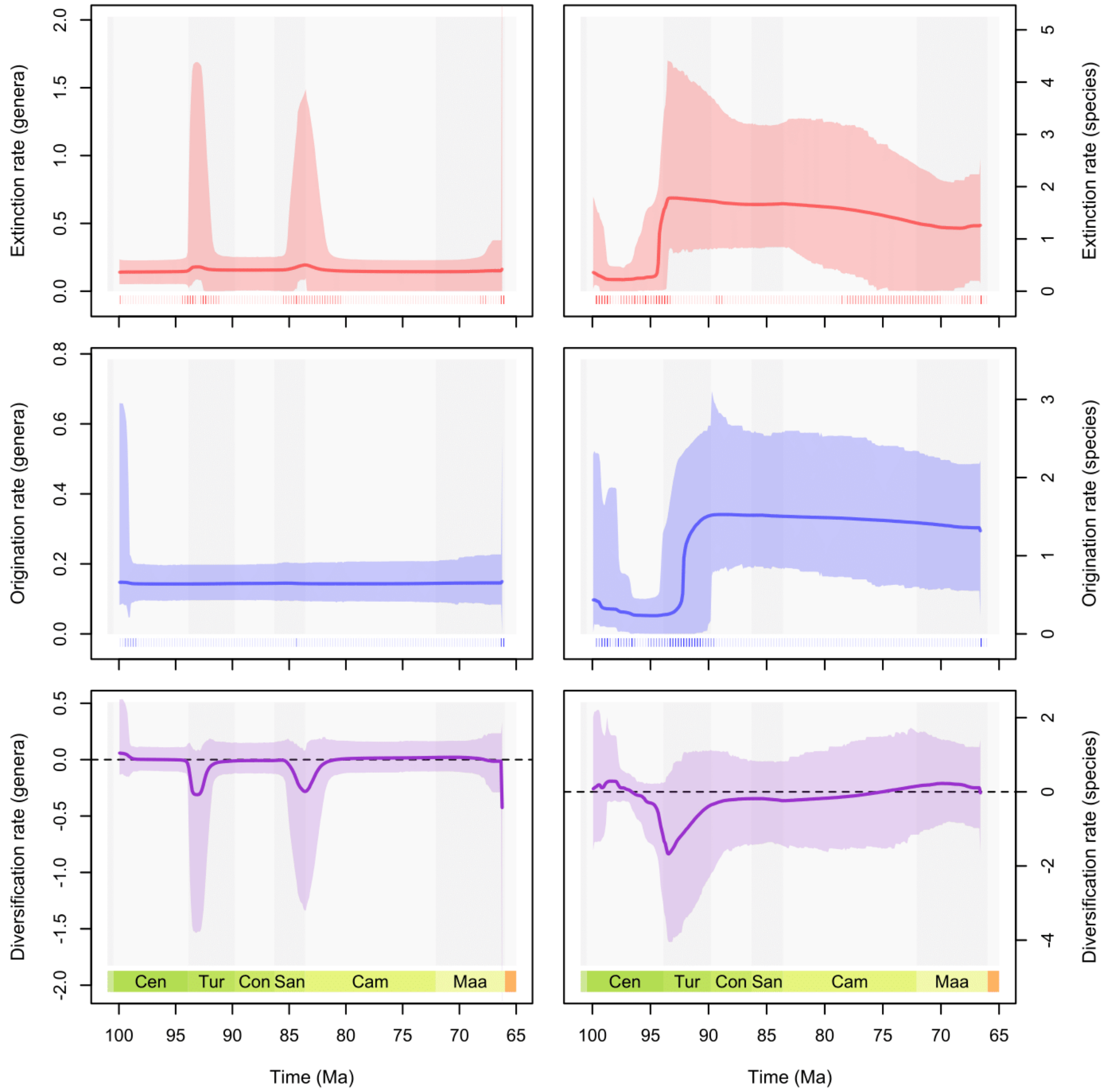

Supplementary Fig. 10. Antarctic Late Cretaceous ammonoid diversification rates. Each plot displays the mean and 95% highest posterior density for each rate estimate. Source data is available in the electronic supplement accompanying this paper.

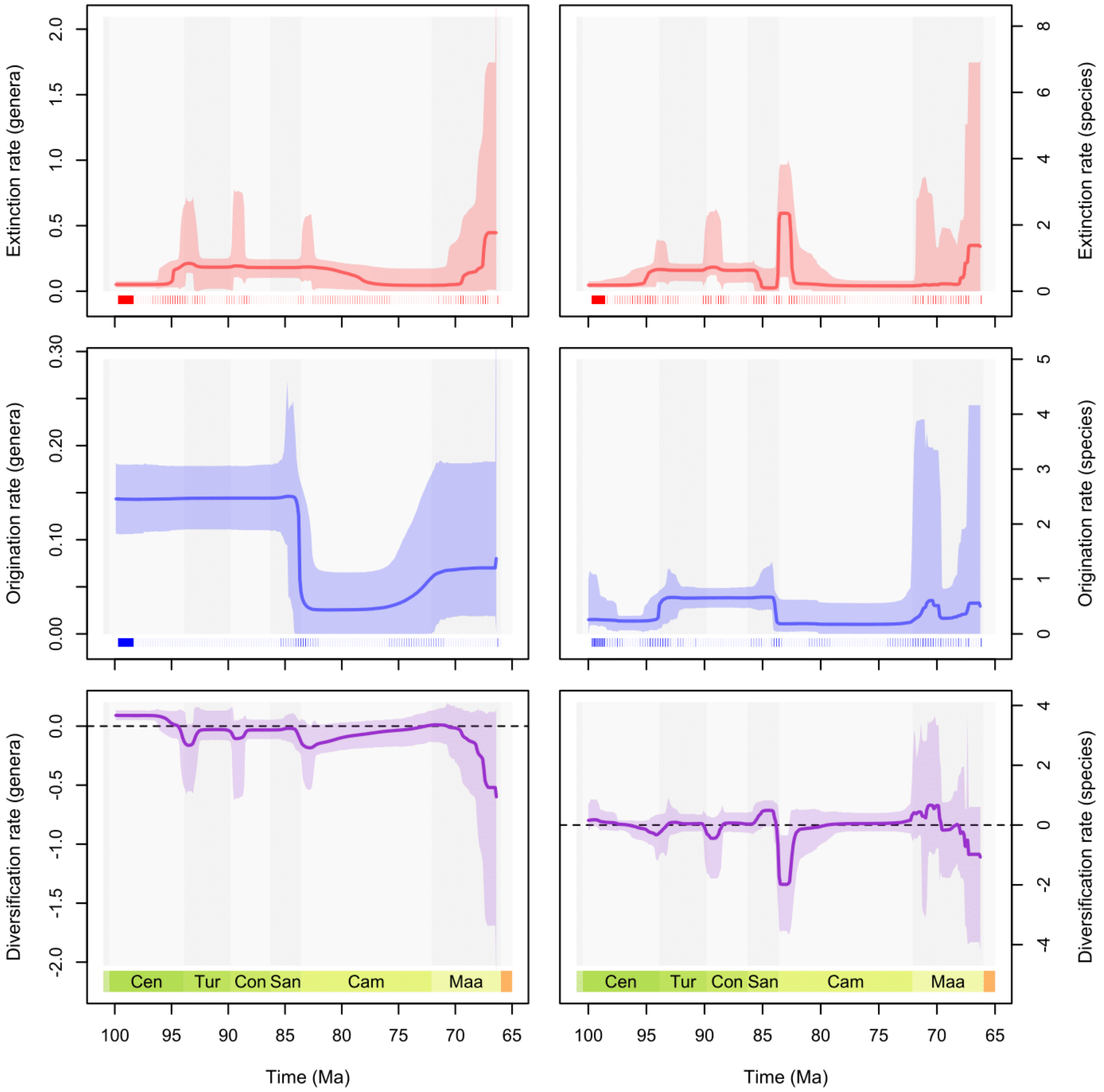

Supplementary Fig. 11. East Pacific Late Cretaceous ammonoid diversification rates. Each plot displays the mean and 95% highest posterior density for each rate estimate. Source data is available in the electronic supplement accompanying this paper.

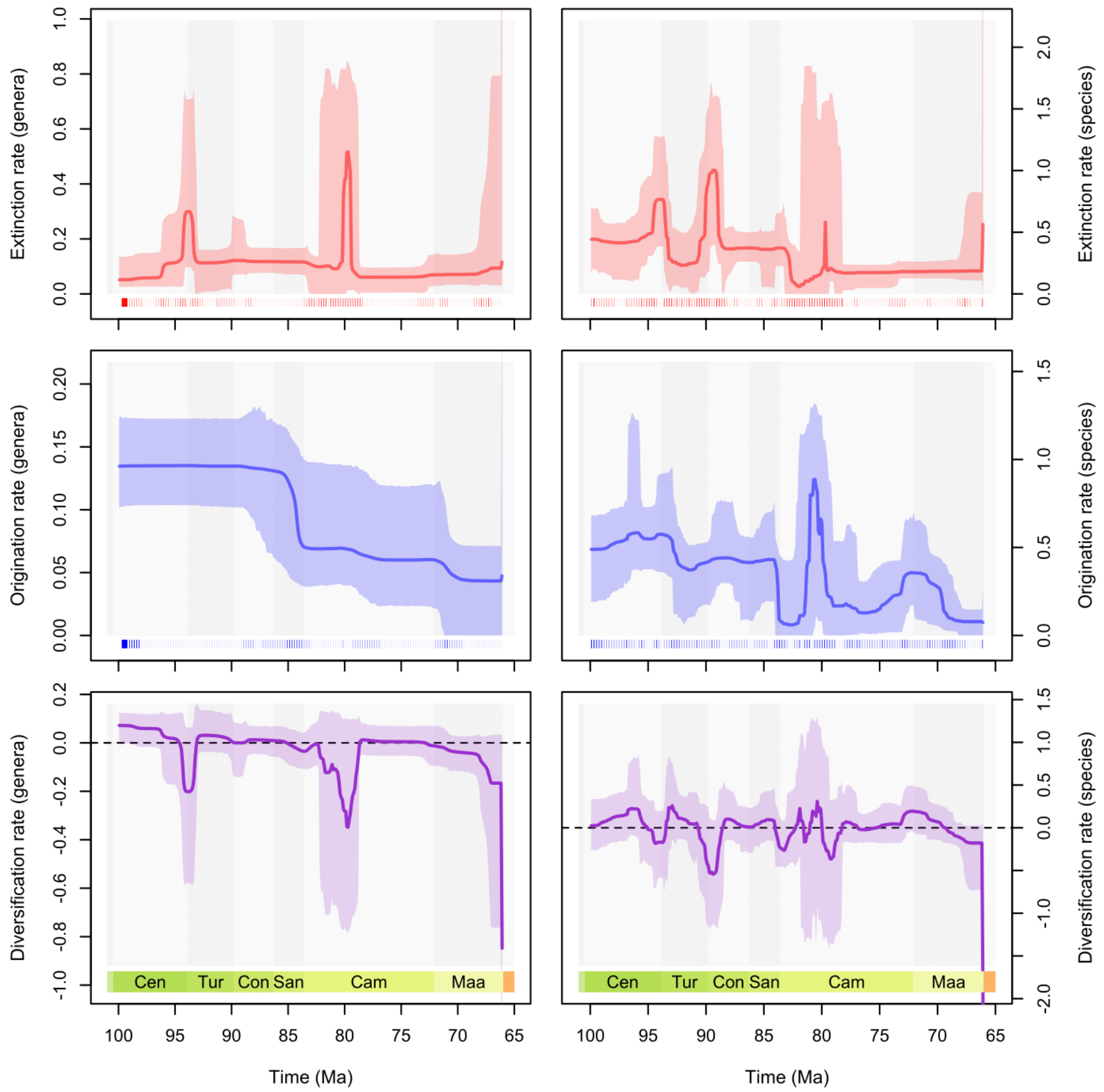

Supplementary Fig. 12. Atlantic and Gulf Late Cretaceous ammonoid diversification rates. Each plot displays the mean and 95% highest posterior density for each rate estimate. Source data is available in the electronic supplement accompanying this paper.

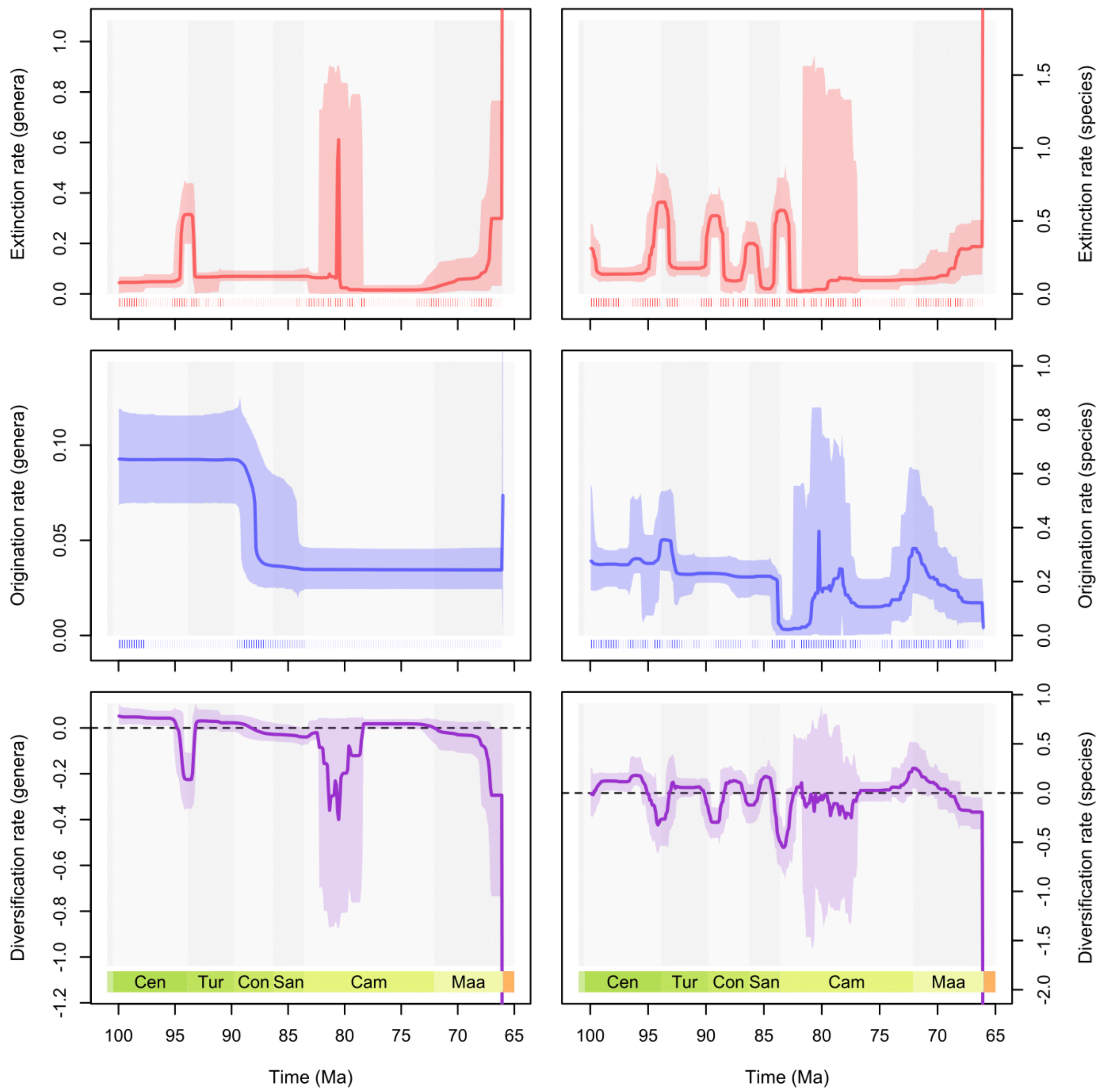

Supplementary Fig. 13. Global Late Cretaceous ammonoid diversification rates. Each plot displays the mean and 95% highest posterior density for each rate estimate. Source data is available in the electronic supplement accompanying this paper.

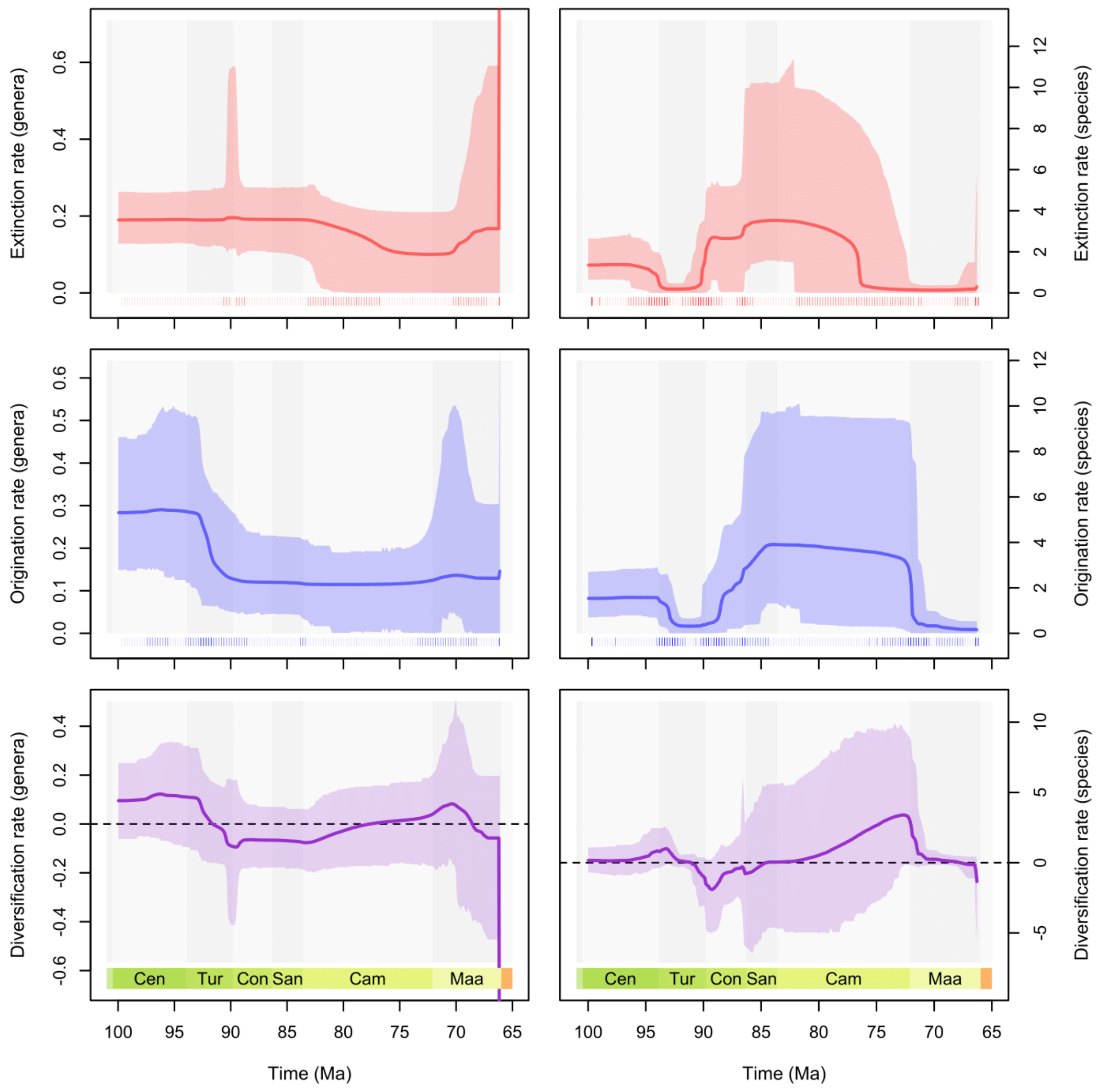

Supplementary Fig. 14. South African Late Cretaceous ammonoid diversification rates. Each plot displays the mean and 95% highest posterior density for each rate estimate. Source data is available in the electronic supplement accompanying this paper. Source data is available in the electronic supplement accompanying this paper.

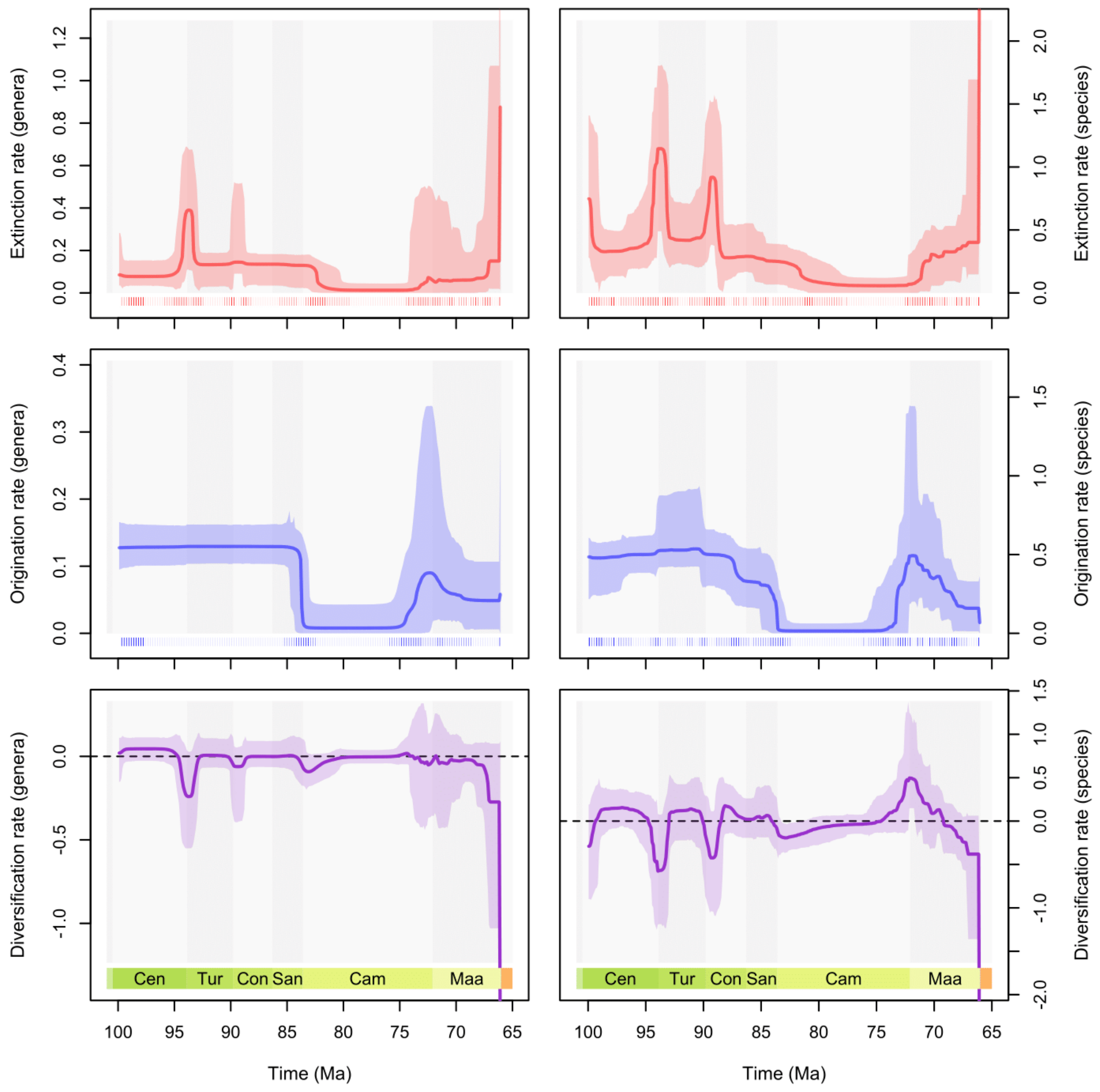

Supplementary Fig. 15. Tethyan Late Cretaceous ammonoid diversification rates. Each plot displays the mean and 95% highest posterior density for each rate estimate. Source data is available in the electronic supplement accompanying this paper.

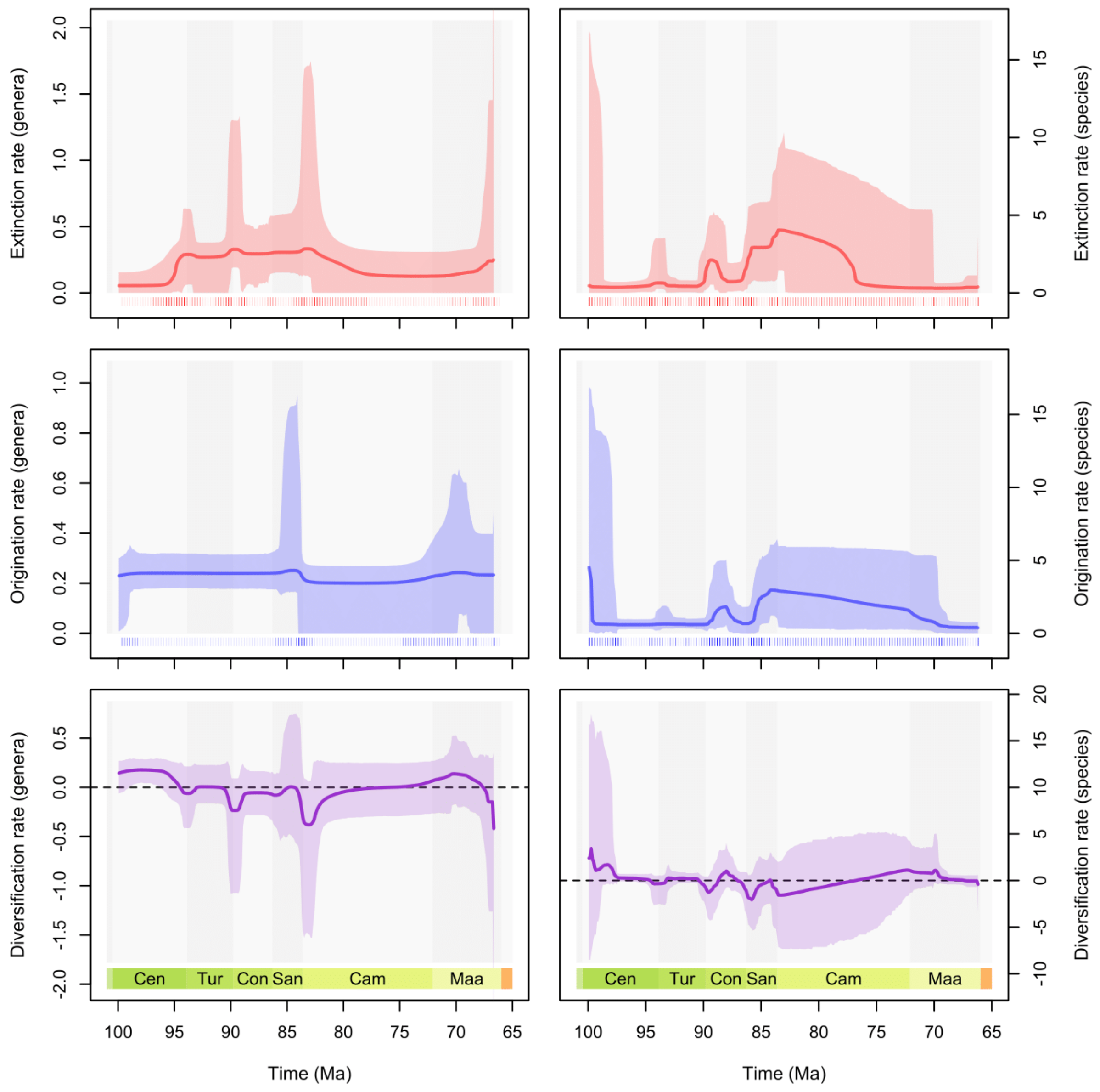

Supplementary Fig. 16. West African Late Cretaceous ammonoid diversification rates. Each plot displays the mean and 95% highest posterior density for each rate estimate. Source data is available in the electronic supplement accompanying this paper.

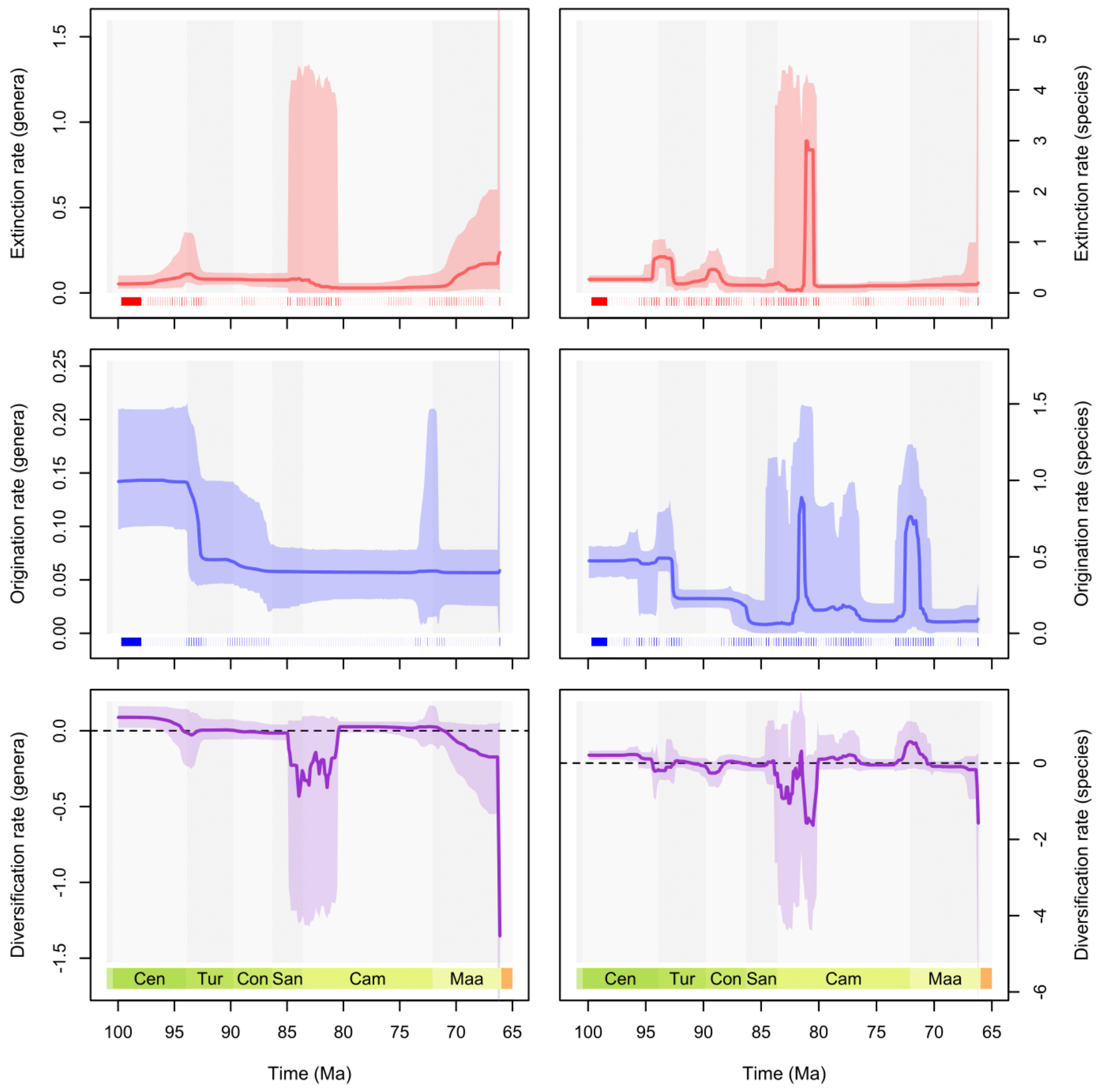

Supplementary Fig. 17. Western Interior Seaway Late Cretaceous ammonoid diversification rates. Each plot displays the mean and 95% highest posterior density for each rate estimate. Source data is available in the electronic supplement accompanying this paper.

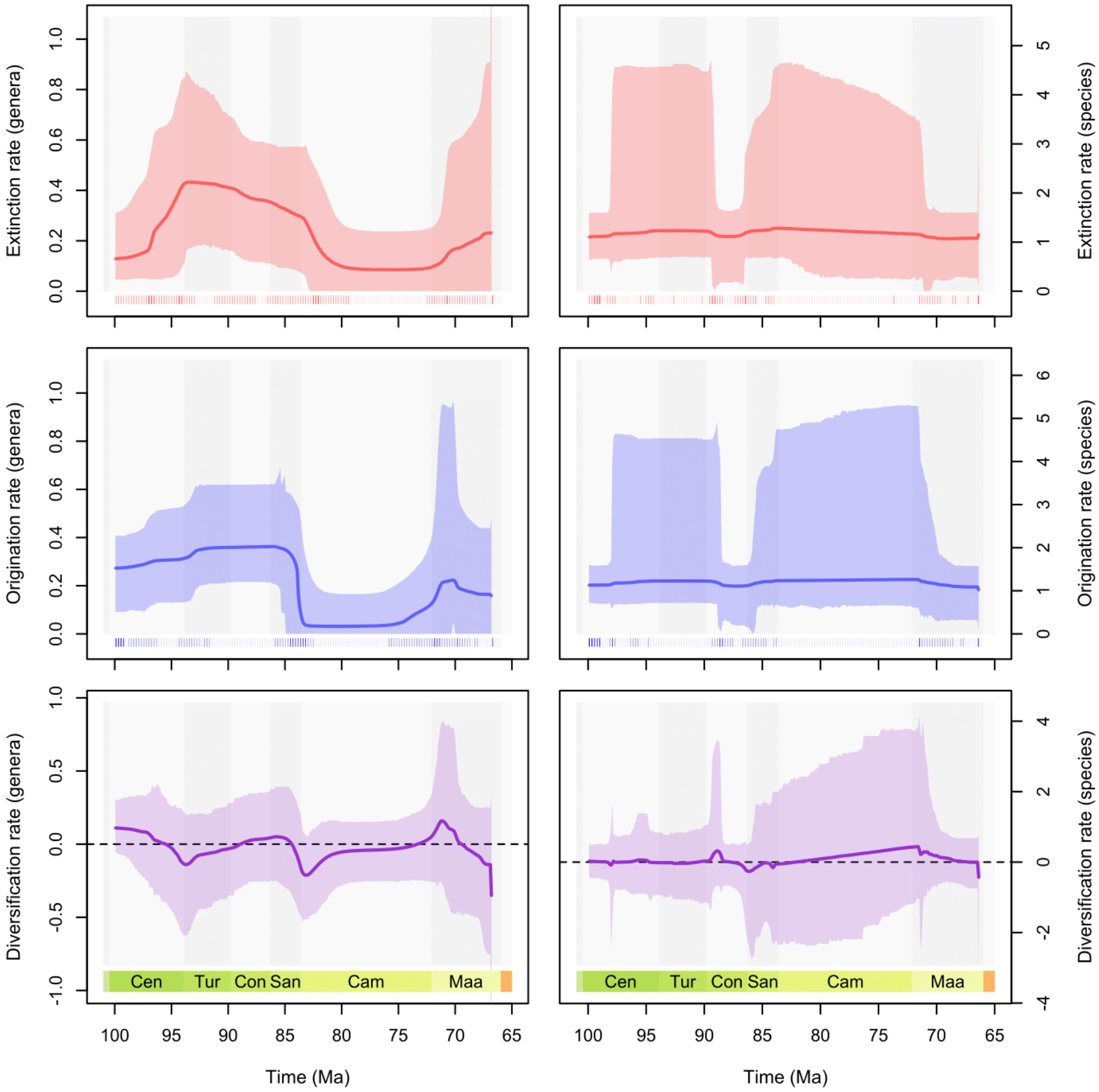

Supplementary Fig. 18. West Pacific Late Cretaceous ammonoid diversification rates. Each plot displays the mean and 95% highest posterior density for each rate estimate. Source data is available in the electronic supplement accompanying this paper.

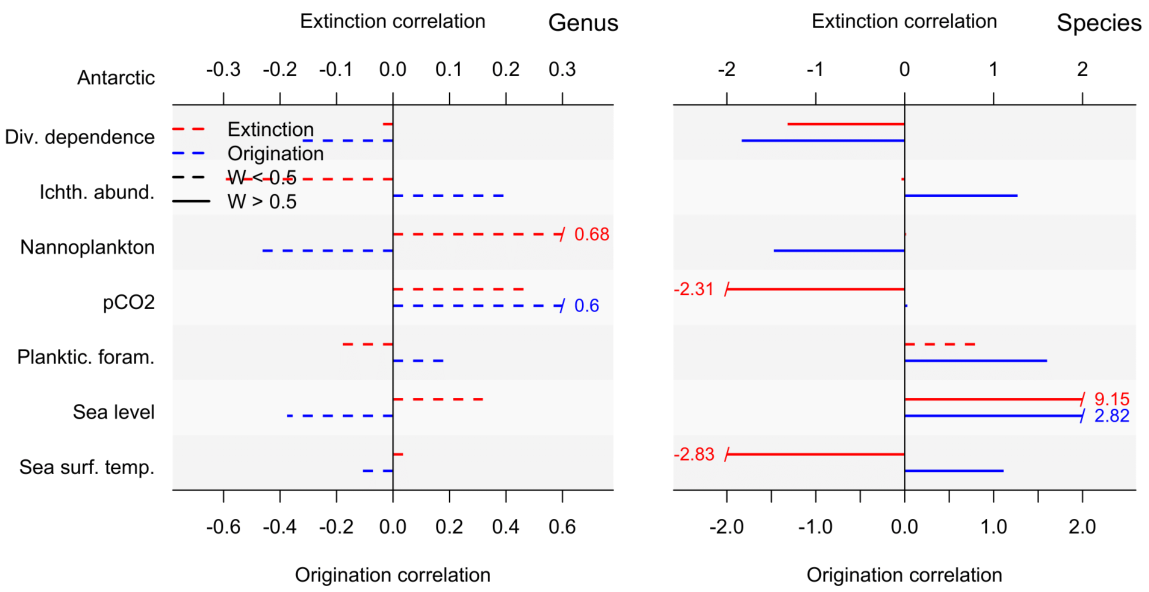

Supplementary Fig. 19. Exponential multivariate birth death correlations for Antarctic ammonoids. W refers to the shrinkage weight of the correlation where W > 0.5 is statistically significant. Div. = diversity; ichth. = ichthyolith; surf. – surface. Source data is available in the electronic supplement accompanying this paper.

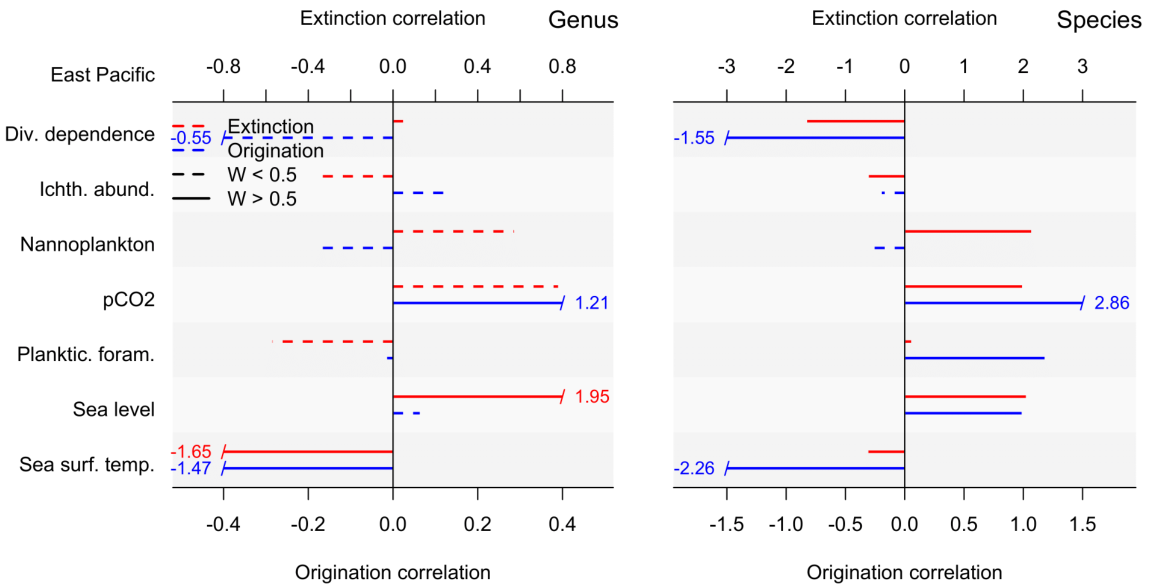

Supplementary Fig. 20. Exponential multivariate birth death correlations for East Pacific ammonoids. W refers to the shrinkage weight of the correlation where W > 0.5 is statistically significant. Div. = diversity; ichth. = ichthyolith; surf. – surface. Source data is available in the electronic supplement accompanying this paper.

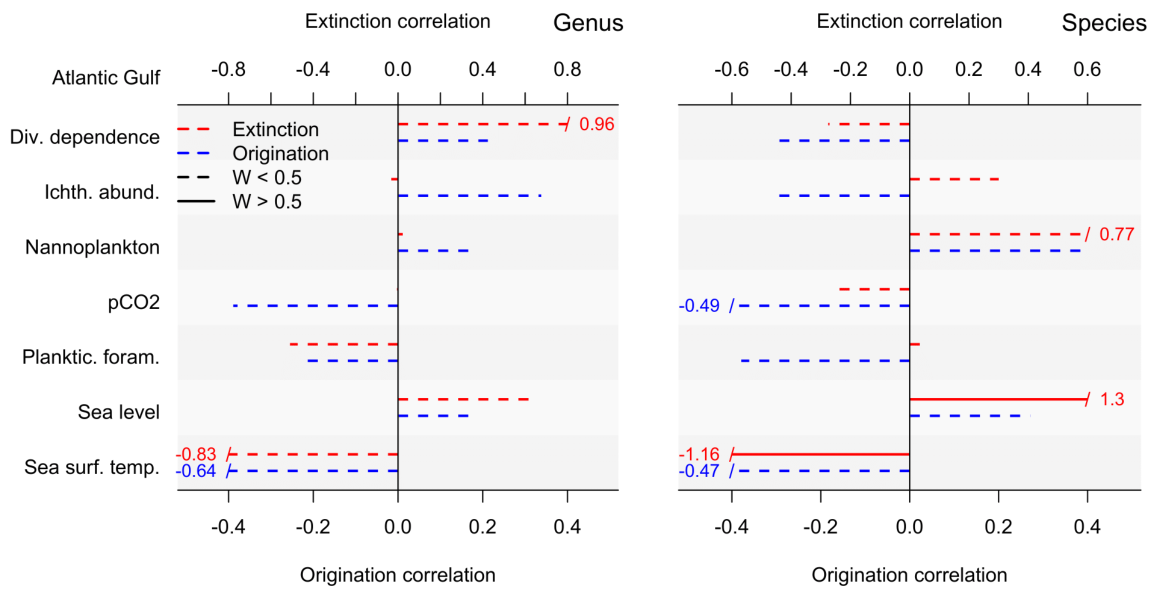

Supplementary Fig. 21. Exponential multivariate birth death correlations for Atlantic and Gulf ammonoids. W refers to the shrinkage weight of the correlation where W > 0.5 is statistically significant. Div. = diversity; ichth. = ichthyolith; surf. – surface. Source data is available in the electronic supplement accompanying this paper.

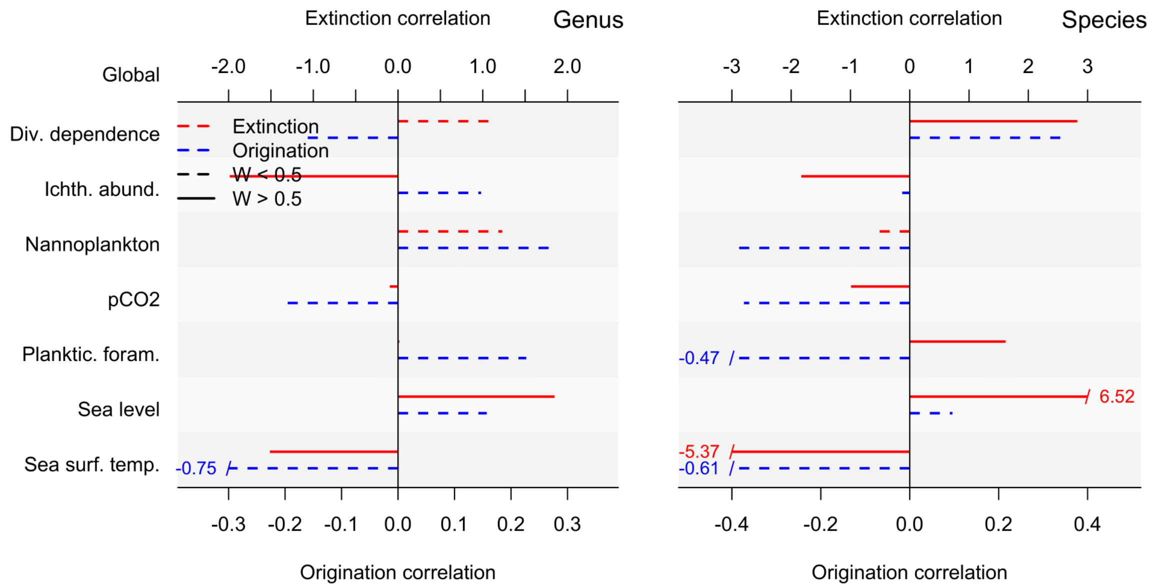

Supplementary Fig. S22. Exponential multivariate birth death correlations for ammonoids globally. W refers to the shrinkage weight of the correlation where W > 0.5 is statistically significant. Div. = diversity; ichth. = ichthyolith; surf. - surface. Source data is available in the electronic supplement accompanying this paper.

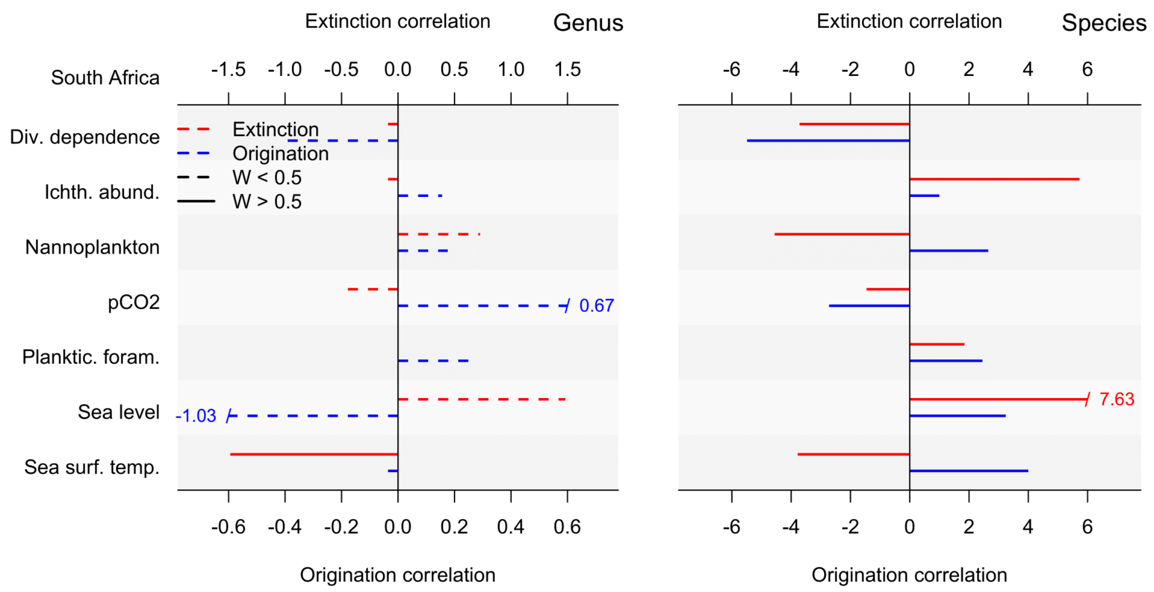

Supplementary Fig. 23. Exponential multivariate birth death correlations for South African ammonoids. W refers to the shrinkage weight of the correlation where W > 0.5 is statistically significant. Div. = diversity; ichth. = ichthyolith; surf. - surface. Source data is available in the electronic supplement accompanying this paper.

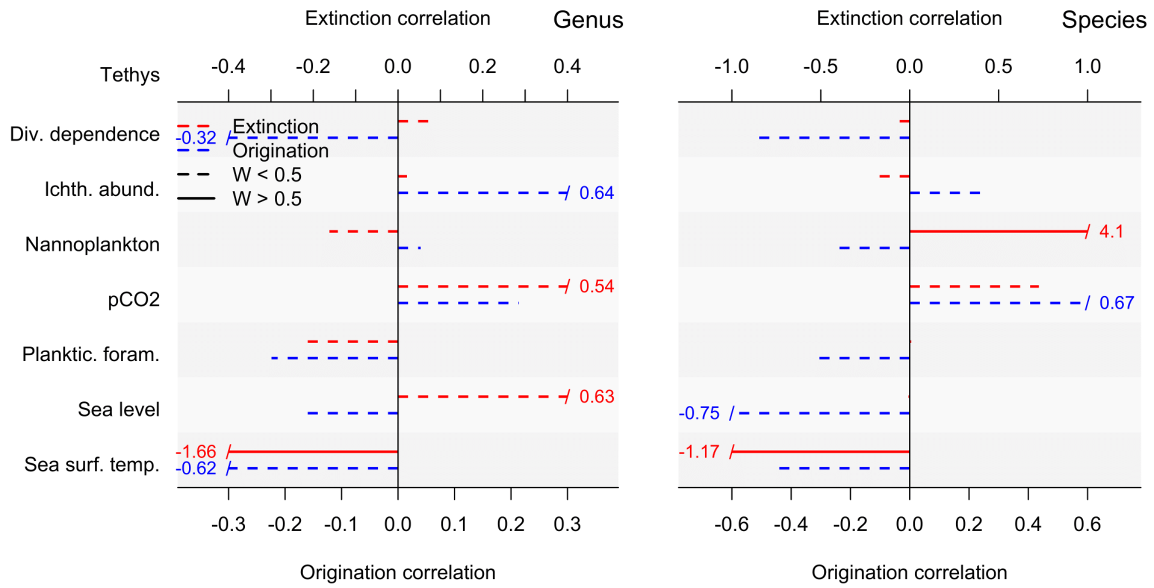

Supplementary Fig. 24. Exponential multivariate birth death correlations for Tethyan ammonoids. W refers to the shrinkage weight of the correlation where W > 0.5 is statistically significant. Div. = diversity; ichth. = ichthyolith; surf. - surface. Source data is available in the electronic supplement accompanying this paper.

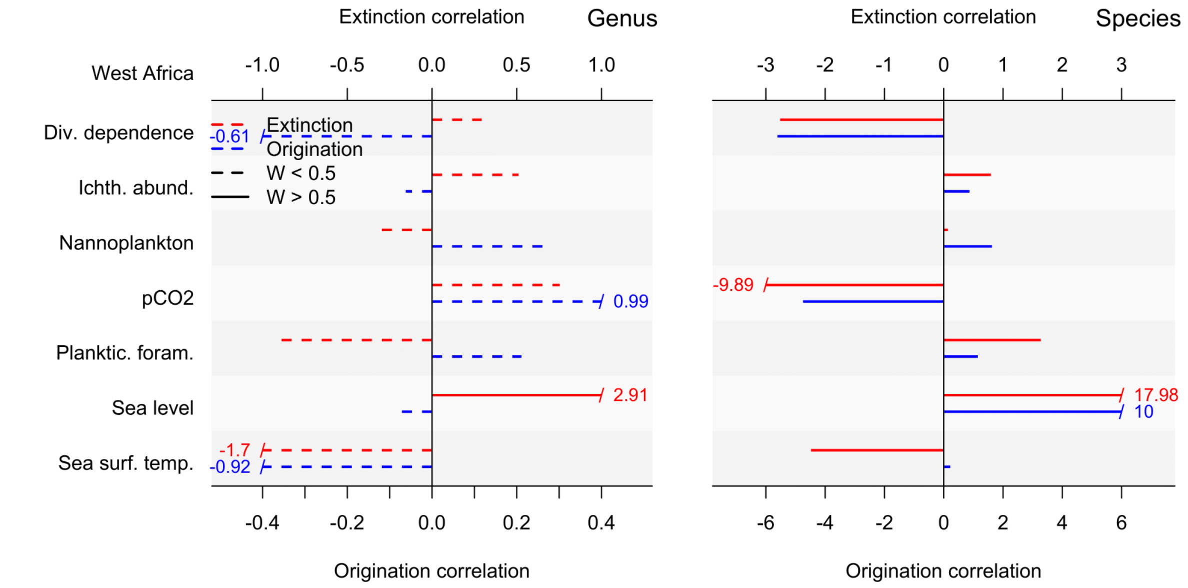

Supplementary Fig. 25. Exponential multivariate birth death correlations for West African ammonoids. W refers to the shrinkage weight of the correlation where W > 0.5 is statistically significant. Div. = diversity; ichth. = ichthyolith; surf. - surface. Source data is available in the electronic supplement accompanying this paper.

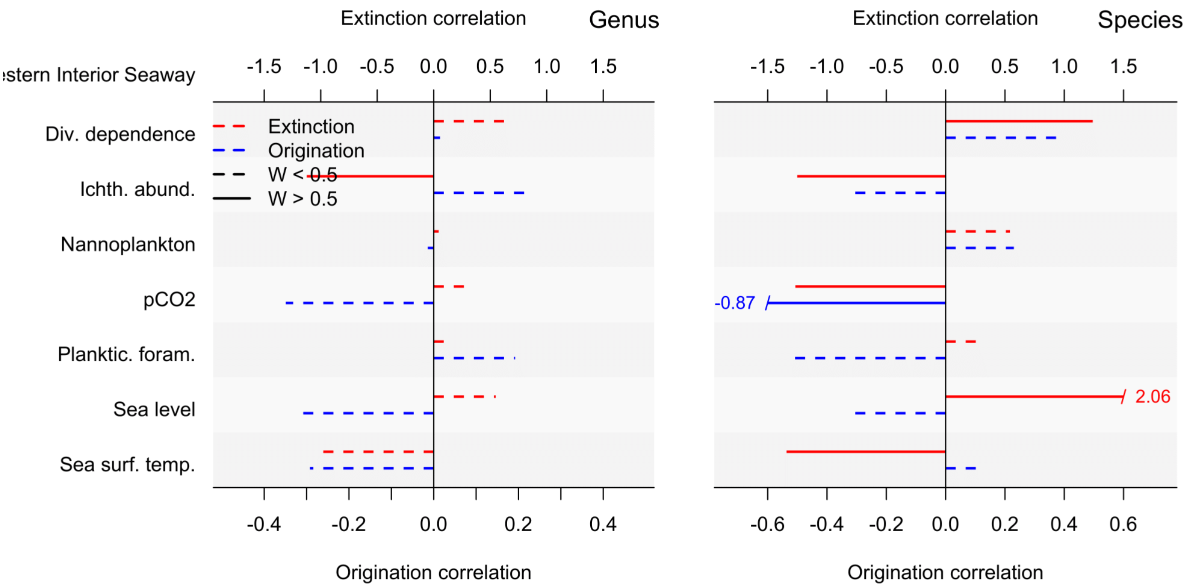

Supplementary Fig. 26. Exponential multivariate birth death correlations for Western Interior Seaway ammonoids. W refers to the shrinkage weight of the correlation where W > 0.5 is statistically significant. Div. = diversity; ichth. = ichthyolith; surf. - surface. Source data is available in the electronic supplement accompanying this paper.

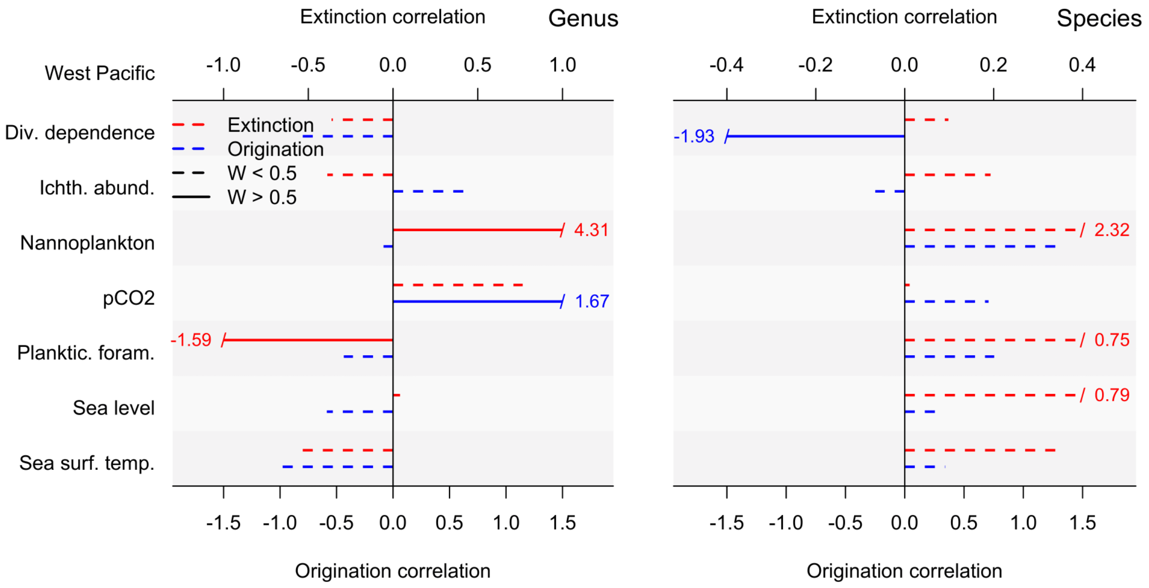

Supplementary Fig. 27. Exponential multivariate birth death correlations for West Pacific ammonoids. W refers to the shrinkage weight of the correlation where W > 0.5 is statistically significant. Div. = diversity; ichth. = ichthyolith; surf. - surface. Source data is available in the electronic supplement accompanying this paper.

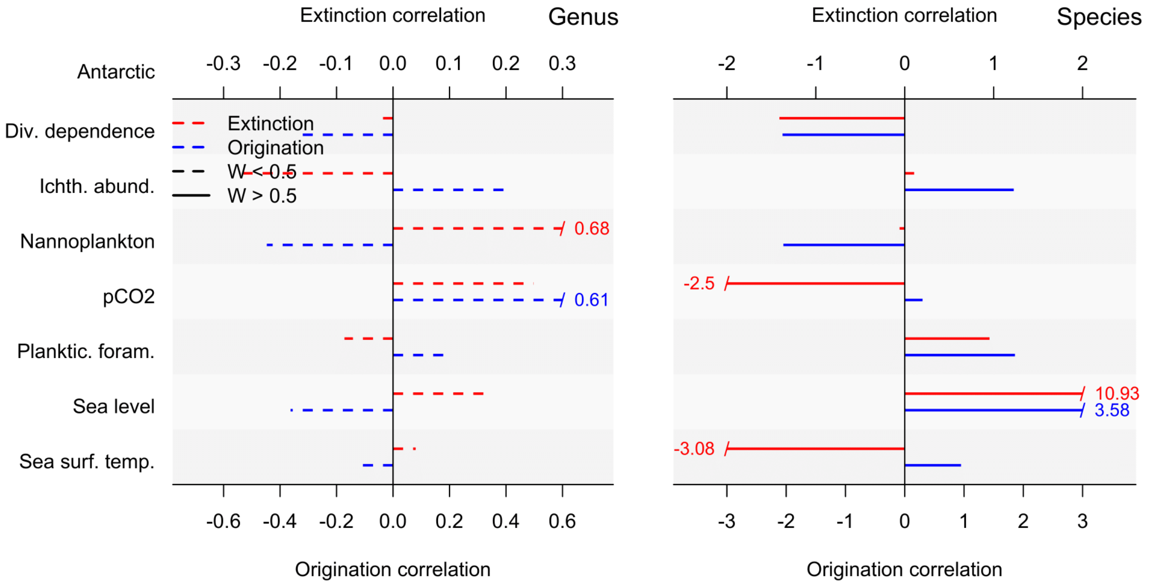

Supplementary Fig. 28. Linear multivariate birth death correlations for Antarctic ammonoids. W refers to the shrinkage weight of the correlation where W > 0.5 is statistically significant. Div. = diversity; ichth. = ichthyolith; surf. – surface. Source data is available in the electronic supplement accompanying this paper.

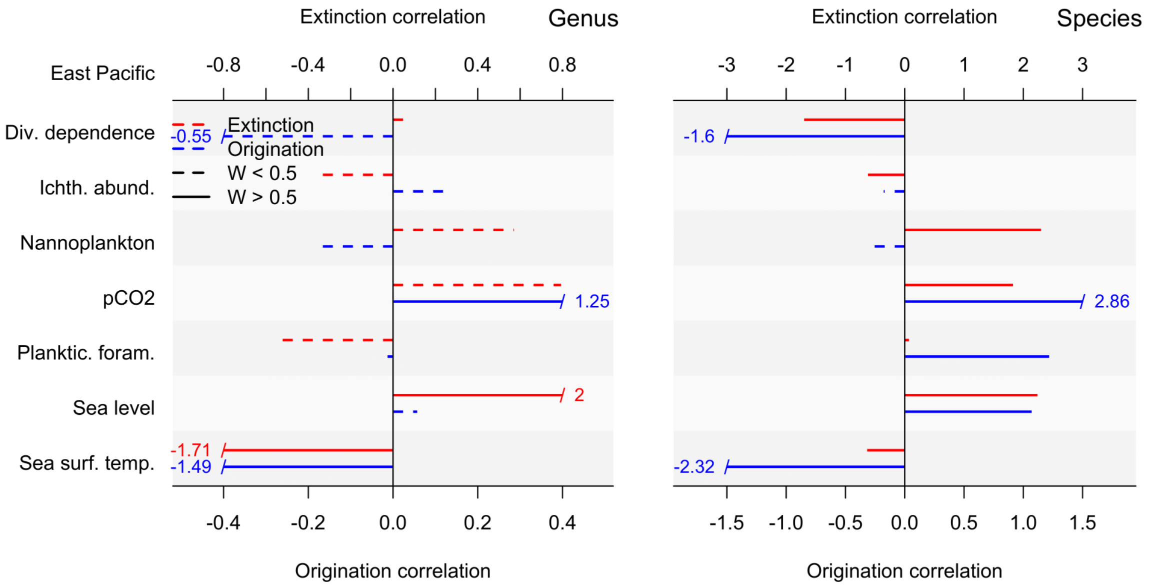

Supplementary Fig. 29. Linear multivariate birth death correlations for East Pacific ammonoids. W refers to the shrinkage weight of the correlation where W > 0.5 is statistically significant. Div. = diversity; ichth. = ichthyolith; surf. – surface. Source data is available in the electronic supplement accompanying this paper.

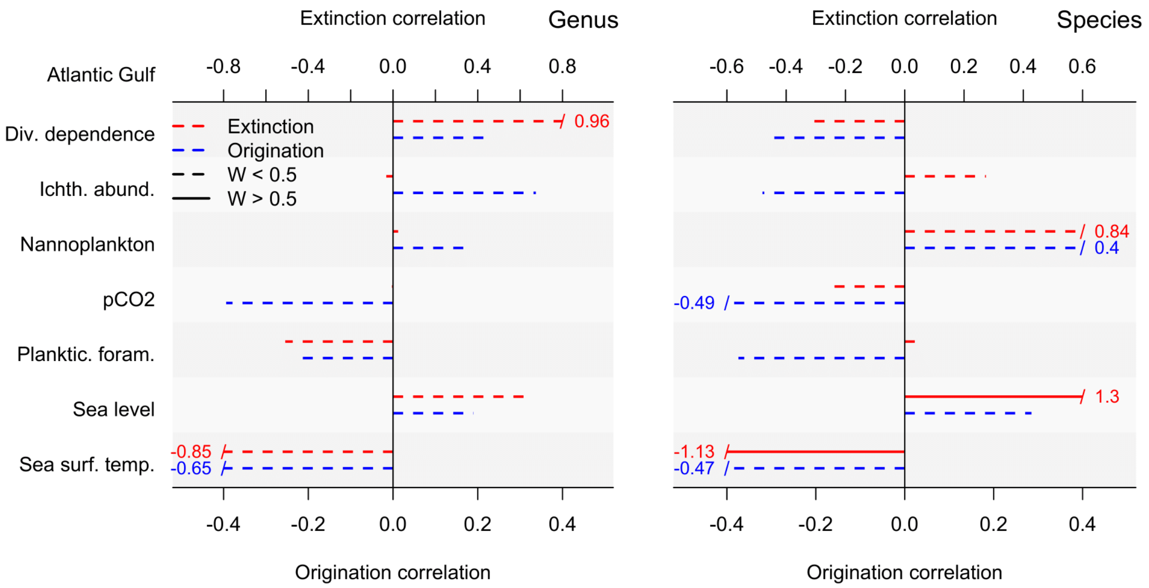

Supplementary Fig. 30. Linear multivariate birth death correlations for Atlantic and Gulf ammonoids. W refers to the shrinkage weight of the correlation where W > 0.5 is statistically significant. Div. = diversity; ichth. = ichthyolith; surf. – surface. Source data is available in the electronic supplement accompanying this paper.

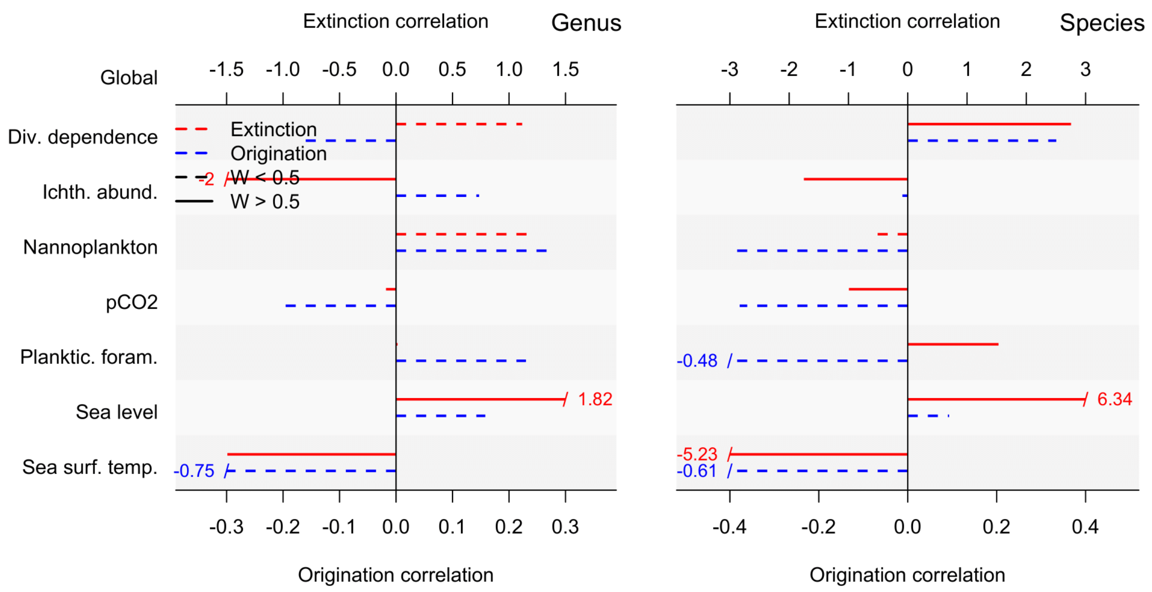

Supplementary Fig. 31. Linear multivariate birth death correlations for ammonoids globally. W refers to the shrinkage weight of the correlation where W > 0.5 is statistically significant. Div. = diversity; ichth. = ichthyolith; surf. - surface. Source data is available in the electronic supplement accompanying this paper.

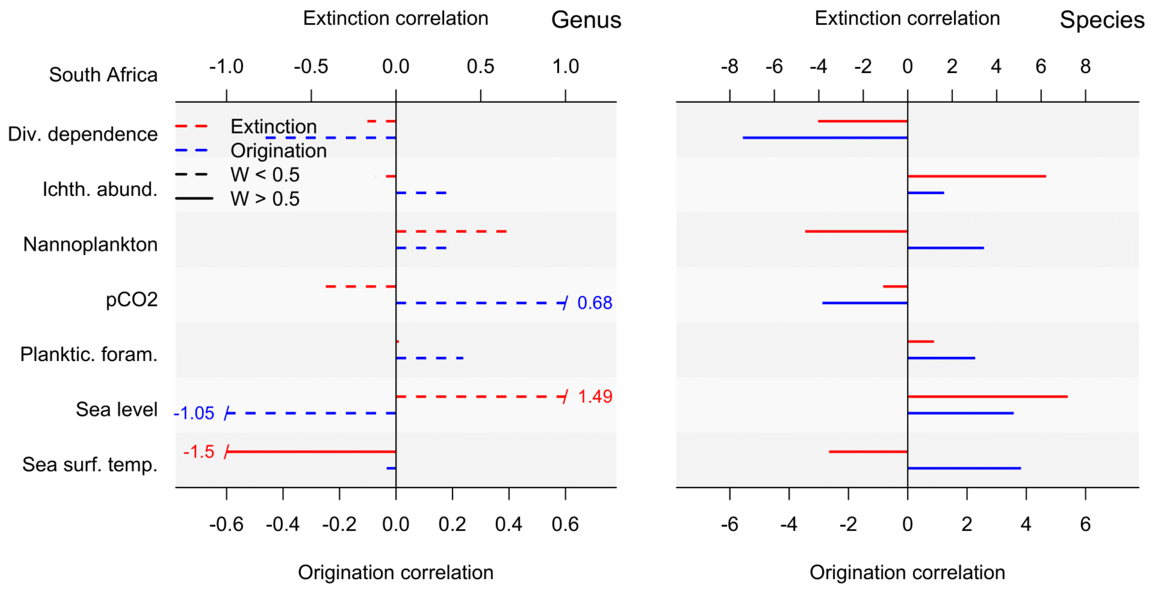

Supplementary Fig. 32. Linear multivariate birth death correlations for South African ammonoids. W refers to the shrinkage weight of the correlation where W > 0.5 is statistically significant. Div. = diversity; ichth. = ichthyolith; surf. – surface. Source data is available in the electronic supplement accompanying this paper.

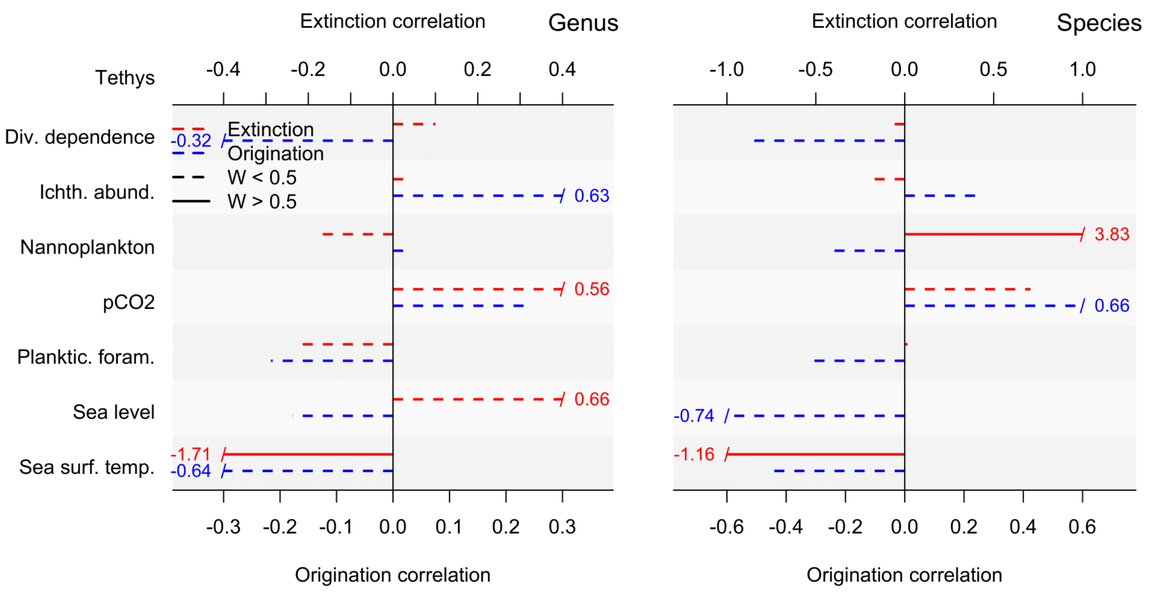

Supplementary Fig. 33. Linear multivariate birth death correlations for Tethyan ammonoids. W refers to the shrinkage weight of the correlation where W > 0.5 is statistically significant. Div. = diversity; ichth. = ichthyolith; surf. - surface. Source data is available in the electronic supplement accompanying this paper.

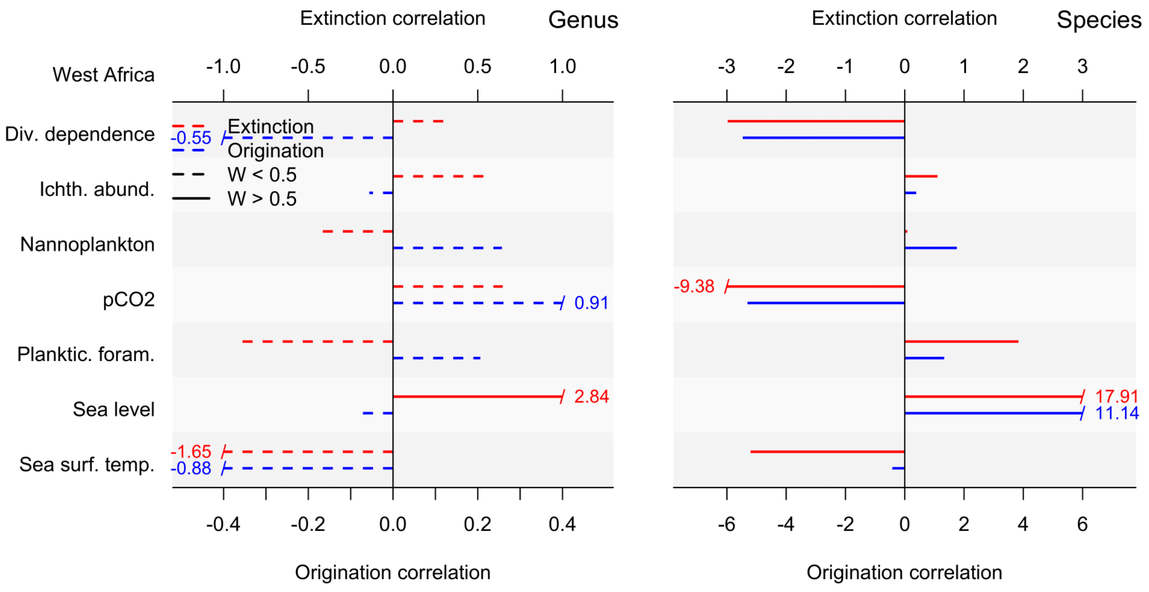

Supplementary Fig. 34. Linear multivariate birth death correlations for West African ammonoids. W refers to the shrinkage weight of the correlation where W > 0.5 is statistically significant. Div. = diversity; ichth. = ichthyolith; surf. - surface. Source data is available in the electronic supplement accompanying this paper.

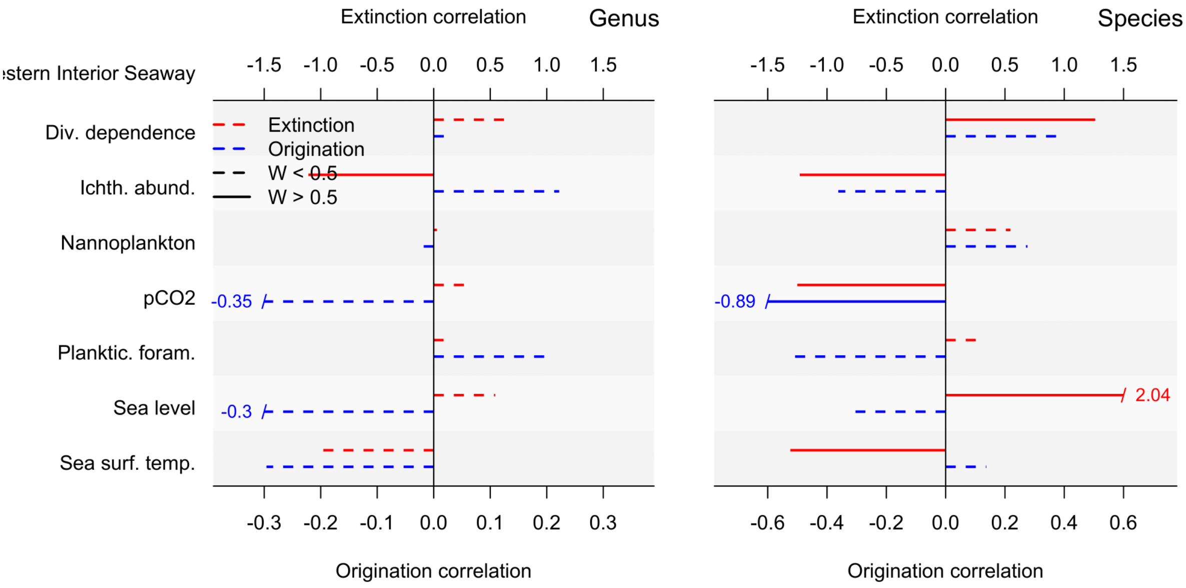

Supplementary Fig. 35. Linear multivariate birth death correlations for Western Interior Seaway ammonoids. W refers to the shrinkage weight of the correlation where W > 0.5 is statistically significant. Div. = diversity; ichth. = ichthyolith; surf. - surface. Source data is available in the electronic supplement accompanying this paper.

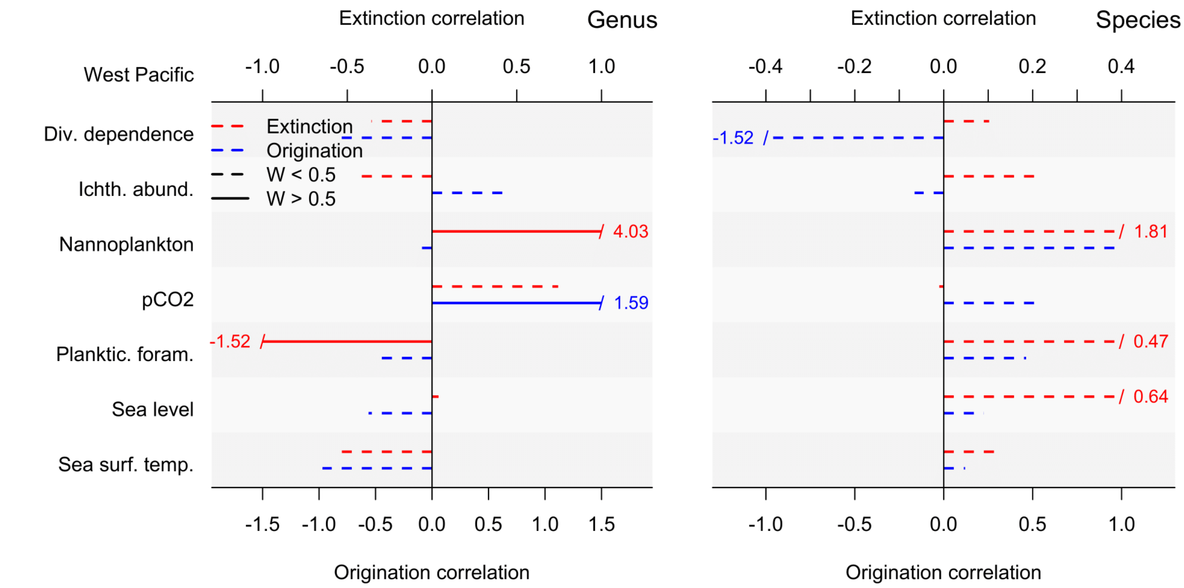

Supplementary Fig. 36. Linear multivariate birth death correlations for West Pacific ammonoids. W refers to the shrinkage weight of the correlation where W > 0.5 is statistically significant. Div. = diversity; ichth. = ichthyolith; surf. - surface. Source data is available in the electronic supplement accompanying this paper.

Supplementary Table 1. Correlations between Late Cretaceous ammonoid spatial extent and subsampled diversity

			Pearson		Spearman
Region	Metric	r	p	ρ	p
Antarctic	MST length	0.664	0.052	0.536	0.118
	Lat range	0.626	0.066	0.357	0.222
	Lng range	0.002	0.498	-0.214	0.703
E. Pacific	MST length	0.276	0.274	0.143	0.391
	Lat range	-0.458	0.849	-0.342	0.774
	Lng range	-0.287	0.734	0.143	0.391
Gulf	MST length	-0.139	0.617	-0.321	0.778
	Lat range	-0.039	0.533	-0.143	0.643
	Lng range	-0.096	0.581	-0.393	0.823
Global	MST length	-0.008	0.507	-0.214	0.703
	Lat range	-0.031	0.527	0.378	0.201
	Lng range	0.366	0.21	0.559	0.096
S. Africa	MST length	-0.533	0.891	-0.464	0.867
	Lat range	-0.424	0.828	-0.342	0.774
	Lng range	0.65	0.057	0.396	0.189
Tethys	MST length	-0.444	0.841	-0.643	0.945
	Lat range	0.059	0.45	0.321	0.249
	Lng range	-0.227	0.688	-0.252	0.707
W. Africa	MST length	0.152	0.373	0.143	0.391
	Lat range	-0.513	0.881	-0.429	0.849
	Lng range	0.375	0.204	0.357	0.222
WIS	MST length	-0.179	0.65	-0.5	0.882
	Lat range	0.388	0.195	0.429	0.177
	Lng range	0.025	0.479	-0.214	0.703
W. Pacific	MST length	-0.208	0.673	-0.767	0.978
	Lat range	-0.032	0.527	0	0.518
	Lng range	-0.068	0.558	-0.143	0.643

Supplementary Table 2. Fits of PyRate preservation models to spatially standardised regional and global Late Cretaceous ammonoid occurrence datasets

Region (taxon)	HPP	NHPP	TPP
Antarctic (g)	1492	1498.4	1400.1
Antarctic (s)	779.4	783	773.6
E. Pacific (g)	6498	6492.6	6317.1
E. Pacific (s)	2313	2256.9	2211.7
Gulf (g)	25140.1	25244.6	24609.9
Gulf (s)	11790	11898.4	11258.3
Global (g)	146851.1	141950	141903.4
Global (s)	78800.8	78565.5	74470.9
S. Africa (g)	1675.2	1653.4	1546.4
S. Africa (s)	670.7	676.6	650.5
Tethys (g)	27166.1	26850.4	26390
Tethys (s)	12305.1	12064.8	11121.9
W. Africa (g)	2689.8	2706.4	2447.1
W. Africa (s)	1025.5	1026.8	997.4
WIS (g)	40673.9	39831.3	36377.2
WIS (s)	20437.7	20775.5	18603.8
W. Pacific (g)	1398.5	1389.2	1285.4
W. Pacific (s)	420.6	427	407.4

g = genus; s = species; HPP = homogenous Poisson process; NHPP = non-homogenous Poisson process; TPP = time-variable piecewise constant Poisson Process; WIS = Western Interior Seaway

Supplementary Table 3. Fits of exponential and linear birth-death correlation models to regional and global Late Cretaceous ammonoid diversification rates

Region (taxon)	AIC (exp)	AIC (lin)	ΔAIC	lnl (exp)	lnl (lin)	log(BF)
Antarctic (g)	586.1	585.68	0.42	-274.58	-274.66	0.16
Antarctic (s)	412.09	412.53	-0.45	-193.71	-193.97	0.52
E. Pacific (g)	1438.04	1438.19	-0.15	-708.39	-708.39	0
E. Pacific (s)	1837.91	1837.5	0.41	-904.27	-904.24	-0.07
Gulf (g)	2209.12	2209.82	-0.7	-1089.99	-1090.03	0.09
Gulf (s)	4733.23	4731.43	1.8	-2350.55	-2350.32	-0.47
Global (g)	3040.38	3040.35	0.04	-1506.86	-1506.88	0.04
Global (s)	12324.62	12324.89	-0.27	-6148.05	-6148.15	0.2
S. Africa (g)	801.63	801.99	-0.36	-385.2	-385.25	0.1
S. Africa (s)	499.19	499.07	0.12	-238.87	-238.75	-0.24
Tethys (g)	2152.92	2153.89	-0.96	-1066.15	-1066.29	0.27
Tethys (s)	4010.98	4009.81	1.17	-1993.93	-1993.78	-0.31
W. Africa (g)	938.27	937.32	0.96	-451.73	-451.67	-0.11
W. Africa (s)	642.15	642.42	-0.27	-309.43	-309.54	0.21
WIS (g)	2193.99	2193.71	0.29	-1084.38	-1084.29	-0.19
WIS (s)	5398.15	5397.22	0.93	-2687.46	-2687.3	-0.32
W. Pacific (g)	746.18	747.13	-0.95	-362.19	-362.33	0.28
W. Pacific (s)	352.85	352.9	-0.05	-167.61	-167.74	0.26

g = genus; s = species; exp = exponential; lin = linear; WIS = Western Interior Seaway

Supplementary Table 4. Late Cretaceous Antarctic ammonoid diversification drivers

	W (sp)	l95 (sp)	u95 (sp)	W (ex)	l95 (ex)	u95 (ex)
Exponential (g)						
Diversity dep.	0.207	-0.333	-1.632	0.397	0.155	-0.025
Ichthyolith abund.	0.216	0.422	-0.402	2.167	0.203	-0.296
Nannoplankton div.	0.236	-0.467	-2.676	0.576	0.243	0.675
pCO_2	0.243	0.605	-0.483	3.144	0.178	0.244
Planktic foram div.	0.17	0.179	-0.994	1.697	0.16	-0.091
Sea level	0.207	-0.375	-2.635	0.739	0.169	0.159
Sea surface temp.	0.168	-0.125	-1.44	0.7	0.172	0.028
Linear (g)						
Diversity dep.	0.205	-0.324	-1.651	0.396	0.154	-0.021
Ichthyolith abund.	0.216	0.415	-0.38	2.143	0.202	-0.282
Nannoplankton div.	0.234	-0.447	-2.627	0.624	0.243	0.683
pCO_2	0.243	0.607	-0.471	3.14	0.178	0.249
Planktic foram div.	0.171	0.188	-0.971	1.803	0.159	-0.086
Sea level	0.205	-0.363	-2.627	0.736	0.169	0.166
Sea surface temp.	0.166	-0.123	-1.425	0.727	0.172	0.04
Exponential (s)						
Diversity dep.	0.599	-1.833	-6.498	0.707	0.577	-1.317
Ichthyolith abund.	0.538	1.269	-2.168	6.765	0.453	-0.037
Nannoplankton div.	0.58	-1.472	-6.095	1.562	0.526	0.015
pCO_2	0.473	0.029	-3.822	4.23	0.592	-2.313
Planktic foram div.	0.537	1.601	-2.391	8.505	0.463	0.849
Sea level	0.64	2.821	-1.599	11.329	0.881	9.155
Sea surface temp.	0.563	1.112	-4.896	9.351	0.627	-2.828
Linear (s)						
Diversity dep.	0.638	-2.061	-7.628	1.147	0.607	-1.409
Ichthyolith abund.	0.573	1.838	-2.799	9.916	0.49	0.105
Nannoplankton div.	0.602	-2.048	-9.811	1.995	0.557	-0.058
pCO_2	0.521	0.3	-4.403	5.878	0.619	-2.501
Planktic foram div.	0.572	1.859	-3.21	10.444	0.506	0.953
Sea level	0.687	3.58	-1.698	13.994	0.896	10.932
Sea surface temp.	0.595	0.949	-7.599	10.506	0.656	-3.085

g = genus; s = species; ex = extinction; sp = speciation; W = correlation shrinkage weight

Supplementary Table 5. Late Cretaceous East Pacific ammonoid diversification drivers

	W (sp)	l95 (sp)	u95 (sp)	W (ex)	l95 (ex)	u95 (ex)
Exponential (g)						
Diversity dep.	0.368	-0.549	-1.736	0.37	0.292	0.078
Ichthyolith abund.	0.219	0.121	-0.501	0.853	0.346	-0.338
Nannoplankton div.	0.28	-0.181	-1.21	0.703	0.357	0.571
pCO_2	0.51	1.213	-0.233	3.447	0.466	0.779
Planktic foram div.	0.28	-0.014	-1.169	1.187	0.391	-0.57
Sea level	0.249	0.063	-0.849	1.074	0.54	1.952
Sea surface temp.	0.635	-1.472	-2.901	-0.449	0.532	-1.654
Linear (g)						
Diversity dep.	0.368	-0.549	-1.779	0.366	0.289	0.055
Ichthyolith abund.	0.22	0.118	-0.529	0.85	0.35	-0.36
Nannoplankton div.	0.282	-0.172	-1.213	0.733	0.359	0.571
pCO_2	0.511	1.245	-0.246	3.652	0.467	0.793
Planktic foram div.	0.285	-0.013	-1.144	1.22	0.39	-0.549
Sea level	0.248	0.057	-0.841	1.088	0.545	2.003
Sea surface temp.	0.635	-1.487	-2.933	-0.389	0.536	-1.706
Exponential (s)						
Diversity dep.	0.648	-1.554	-4.088	-0.228	0.644	-1.646
Ichthyolith abund.	0.359	-0.194	-1.506	0.971	0.508	-0.606
Nannoplankton div.	0.396	-0.321	-1.765	0.876	0.609	2.132
pCO_2	0.706	2.858	-0.836	9.13	0.603	1.978
Planktic foram div.	0.501	1.179	-0.671	4.548	0.474	0.108
Sea level	0.532	0.986	-1.874	4.921	0.59	2.043
Sea surface temp.	0.707	-2.257	-6.126	0.313	0.544	-0.613
Linear (s)						
Diversity dep.	0.643	-1.6	-4.232	-0.153	0.637	-1.697
Ichthyolith abund.	0.358	-0.178	-1.492	1.062	0.504	-0.62
Nannoplankton div.	0.394	-0.319	-1.769	0.879	0.597	2.297
pCO_2	0.695	2.859	-0.84	9.343	0.597	1.826
Planktic foram div.	0.509	1.218	-0.788	4.565	0.473	0.071
Sea level	0.526	1.07	-1.669	5.095	0.589	2.238
Sea surface temp.	0.595	0.949	-7.599	10.506	0.656	-3.085

g = genus; s = species; ex = extinction; sp = speciation; W = correlation shrinkage weight

Supplementary Table 6. Late Cretaceous Atlantic and Gulf ammonoid diversification drivers

	W (sp)	l95 (sp)	u95 (sp)	W (ex)	l95 (ex)	u95 (ex)
Exponential (g)						
Diversity dep.	0.222	0.212	-0.879	1.59	0.374	0.961
Ichthyolith abund.	0.244	0.338	-0.397	1.432	0.267	-0.032
Nannoplankton div.	0.234	0.169	-0.858	1.521	0.312	0.022
pCO_2	0.288	-0.389	-1.29	0.362	0.248	-0.005
Planktic foram div.	0.258	-0.216	-1.187	0.632	0.32	-0.51
Sea level	0.23	0.181	-0.892	1.617	0.325	0.625
Sea surface temp.	0.361	-0.636	-1.874	0.274	0.386	-0.831
Linear (g)						
Diversity dep.	0.221	0.22	-0.883	1.603	0.374	0.963
Ichthyolith abund.	0.243	0.337	-0.377	1.469	0.267	-0.032
Nannoplankton div.	0.233	0.166	-0.873	1.495	0.309	0.024
pCO_2	0.288	-0.394	-1.292	0.354	0.247	-0.005
Planktic foram div.	0.259	-0.213	-1.216	0.609	0.319	-0.509
Sea level	0.234	0.19	-0.932	1.694	0.327	0.652
Sea surface temp.	0.362	-0.647	-1.935	0.292	0.387	-0.849
Exponential (s)						
Diversity dep.	0.265	-0.293	-1.429	0.408	0.408	-0.275
Ichthyolith abund.	0.255	-0.31	-1.519	0.365	0.477	0.3
Nannoplankton div.	0.312	0.389	-0.535	2.106	0.477	0.772
pCO_2	0.352	-0.489	-0.829	0.001	0.301	-0.242
Planktic foram div.	0.32	-0.379	-0.801	0.175	0.249	0.052
Sea level	0.242	0.271	-0.235	1.096	0.529	1.301
Sea surface temp.	0.342	-0.468	-0.912	0.045	0.518	-1.159
Linear (s)						
Diversity dep.	0.268	-0.301	-1.503	0.44	0.409	-0.318
Ichthyolith abund.	0.257	-0.32	-1.603	0.364	0.477	0.274
Nannoplankton div.	0.313	0.401	-0.56	2.176	0.481	0.835
pCO_2	0.354	-0.494	-0.85	0.004	0.305	-0.244
Planktic foram div.	0.33	-0.374	-0.823	0.255	0.246	0.042
Sea level	0.247	0.285	-0.256	1.191	0.529	1.301
Sea surface temp.	0.345	-0.473	-0.927	0.045	0.524	-1.127

g = genus; s = species; ex = extinction; sp = speciation; W = correlation shrinkage weight

Supplementary Table 7. Late Cretaceous global ammonoid diversification drivers

	W (sp)	l95 (sp)	u95 (sp)	W (ex)	l95 (ex)	u95 (ex)
Exponential (g)						
Diversity dep.	0.265	-0.173	-1.386	0.811	0.393	1.104
Ichthyolith abund.	0.205	0.147	-0.399	0.868	0.548	-1.986
Nannoplankton div.	0.262	0.277	-0.713	1.79	0.44	1.23
pCO_2	0.234	-0.203	-0.904	0.372	0.295	-0.098
Planktic foram div.	0.235	0.227	-0.608	1.365	0.257	0.016
Sea level	0.244	0.157	-0.733	1.202	0.564	1.846
Sea surface temp.	0.433	-0.747	-1.762	0.181	0.522	-1.512
Linear (g)						
Diversity dep.	0.266	-0.172	-1.397	0.808	0.39	1.116
Ichthyolith abund.	0.204	0.147	-0.413	0.841	0.547	-1.997
Nannoplankton div.	0.262	0.279	-0.729	1.756	0.439	1.229
pCO_2	0.233	-0.203	-0.937	0.357	0.293	-0.104
Planktic foram div.	0.233	0.23	-0.591	1.396	0.256	0.013
Sea level	0.242	0.158	-0.702	1.203	0.558	1.821
Sea surface temp.	0.433	-0.748	-1.774	0.166	0.517	-1.493
Exponential (s)						
Diversity dep.	0.342	0.339	-0.167	0.93	0.726	2.826
Ichthyolith abund.	0.252	-0.017	-0.743	0.441	0.631	-1.83
Nannoplankton div.	0.419	-0.388	-1.004	0.553	0.457	-0.639
pCO_2	0.37	-0.373	-0.704	0.018	0.546	-0.991
Planktic foram div.	0.416	-0.469	-0.873	0.041	0.578	1.617
Sea level	0.227	0.096	-0.256	0.537	0.931	6.523
Sea surface temp.	0.47	-0.609	-0.893	-0.33	0.909	-5.371
Linear (s)						
Diversity dep.	0.34	0.334	-0.161	0.92	0.724	2.754
Ichthyolith abund.	0.25	-0.012	-0.682	0.443	0.628	-1.752
Nannoplankton div.	0.415	-0.395	-1	0.457	0.458	-0.641
pCO_2	0.372	-0.378	-0.709	0.01	0.543	-0.992
Planktic foram div.	0.414	-0.477	-0.863	0.031	0.576	1.53
Sea level	0.224	0.093	-0.236	0.533	0.931	6.341
Sea surface temp.	0.47	-0.61	-0.875	-0.336	0.907	-5.227

g = genus; s = species; ex = extinction; sp = speciation; W = correlation shrinkage weight

Supplementary Table 8. Late Cretaceous South African ammonoid diversification drivers

	W (sp)	l95 (sp)	u95 (sp)	W (ex)	l95 (ex)	u95 (ex)
Exponential (g)						
Diversity dep.	0.305	-0.424	-2.329	0.771	0.274	-0.142
Ichthyolith abund.	0.245	0.156	-0.981	1.601	0.253	-0.115
Nannoplankton div.	0.231	0.176	-0.876	1.559	0.328	0.726
pCO_2	0.327	0.667	-0.698	3.136	0.346	-0.46
Planktic foram div.	0.251	0.275	-1.05	2.11	0.234	-0.004
Sea level	0.467	-1.032	-3.081	0.424	0.445	1.48
Sea surface temp.	0.236	-0.059	-1.226	1.039	0.515	-1.485
Linear (g)						
Diversity dep.	0.307	-0.463	-2.627	0.746	0.269	-0.169
Ichthyolith abund.	0.248	0.192	-0.866	1.71	0.253	-0.119
Nannoplankton div.	0.232	0.181	-0.901	1.538	0.324	0.71
pCO_2	0.33	0.675	-0.667	3.096	0.347	-0.435
Planktic foram div.	0.242	0.238	-1.05	1.894	0.235	0.017
Sea level	0.465	-1.051	-3.213	0.423	0.447	1.485
Sea surface temp.	0.238	-0.033	-1.17	1.084	0.519	-1.504
Exponential (s)						
Diversity dep.	0.896	-5.487	-14.626	-0.463	0.845	-3.717
Ichthyolith abund.	0.671	0.996	-5.53	8.927	0.818	5.728
Nannoplankton div.	0.715	2.647	-2.852	11.359	0.789	-4.557
pCO_2	0.741	-2.722	-10.829	3.049	0.716	-1.457
Planktic foram div.	0.699	2.452	-2.594	11.001	0.704	1.845
Sea level	0.766	3.24	-7.832	19.165	0.874	7.633
Sea surface temp.	0.756	3.999	-3.436	15.988	0.814	-3.782
Linear (s)						
Diversity dep.	0.894	-5.563	-15.474	-0.412	0.822	-4.032
Ichthyolith abund.	0.666	1.227	-6.785	10.964	0.82	6.221
Nannoplankton div.	0.712	2.571	-2.902	11.49	0.791	-4.613
pCO_2	0.734	-2.88	-12.105	3.348	0.717	-1.103
Planktic foram div.	0.694	2.273	-3.056	10.909	0.69	1.179
Sea level	0.762	3.574	-6.282	18.782	0.869	7.198
Sea surface temp.	0.763	3.82	-3.309	15.354	0.815	-3.538

g = genus; s = species; ex = extinction; sp = speciation; W = correlation shrinkage weight

Supplementary Table 9. Late Cretaceous Tethyan ammonoid diversification drivers

	W (sp)	l95 (sp)	u95 (sp)	W (ex)	l95 (ex)	u95 (ex)
Exponential (g)						
Diversity dep.	0.268	-0.324	-1.284	0.434	0.21	0.075
Ichthyolith abund.	0.27	0.639	-0.512	3.097	0.178	0.021
Nannoplankton div.	0.23	0.04	-0.919	1.406	0.297	-0.162
pCO_2	0.215	0.214	-0.553	1.161	0.302	0.539
Planktic foram div.	0.225	-0.224	-1.073	0.567	0.252	-0.225
Sea level	0.204	-0.172	-1.095	0.468	0.338	0.628
Sea surface temp.	0.372	-0.624	-1.433	0.179	0.628	-1.661
Linear (g)						
Diversity dep.	0.267	-0.318	-1.31	0.42	0.218	0.1
Ichthyolith abund.	0.273	0.631	-0.549	3.081	0.18	0.024
Nannoplankton div.	0.23	0.023	-0.957	1.286	0.299	-0.182
pCO_2	0.223	0.238	-0.519	1.283	0.311	0.565
Planktic foram div.	0.224	-0.216	-1.08	0.568	0.255	-0.217
Sea level	0.209	-0.178	-1.168	0.464	0.347	0.659
Sea surface temp.	0.377	-0.637	-1.476	0.2	0.633	-1.712
Exponential (s)						
Diversity dep.	0.36	-0.522	-1.192	0.053	0.336	-0.073
Ichthyolith abund.	0.241	0.242	-0.145	0.784	0.35	-0.2
Nannoplankton div.	0.25	-0.256	-0.658	0.067	0.676	4.095
pCO_2	0.394	0.672	-0.136	1.848	0.369	0.726
Planktic foram div.	0.317	-0.318	-0.873	0.335	0.358	0.008
Sea level	0.444	-0.749	-1.645	-0.086	0.423	-0.007
Sea surface temp.	0.341	-0.448	-1.009	0.074	0.51	-1.17
Linear (s)						
Diversity dep.	0.355	-0.516	-1.175	0.048	0.335	-0.071
Ichthyolith abund.	0.237	0.24	-0.142	0.772	0.348	-0.192
Nannoplankton div.	0.253	-0.261	-0.657	0.062	0.67	3.826
pCO_2	0.385	0.656	-0.132	1.814	0.365	0.707
Planktic foram div.	0.317	-0.323	-0.871	0.32	0.348	0.015
Sea level	0.44	-0.737	-1.592	-0.07	0.42	-0.003
Sea surface temp.	0.342	-0.453	-1.007	0.057	0.507	-1.163

g = genus; s = species; ex = extinction; sp = speciation; W = correlation shrinkage weight

Supplementary Table 10. Late Cretaceous West African ammonoid diversification drivers

	W (sp)	l95 (sp)	u95 (sp)	W (ex)	l95 (ex)	u95 (ex)
Exponential (g)						
Diversity dep.	0.354	-0.607	-2.729	0.78	0.313	0.293
Ichthyolith abund.	0.226	-0.062	-1.02	0.84	0.327	0.51
Nannoplankton div.	0.264	0.28	-0.911	2	0.326	-0.343
pCO_2	0.406	0.99	-0.451	3.757	0.412	0.753
Planktic foram div.	0.26	0.211	-0.937	1.841	0.441	-0.888
Sea level	0.256	-0.086	-1.462	1.161	0.534	2.911
Sea surface temp.	0.452	-0.919	-2.799	0.446	0.435	-1.7
Linear (g)						
Diversity dep.	0.347	-0.552	-2.483	0.82	0.311	0.333
Ichthyolith abund.	0.219	-0.056	-1.028	0.805	0.324	0.585
Nannoplankton div.	0.261	0.257	-0.913	1.873	0.322	-0.416
pCO_2	0.397	0.912	-0.461	3.321	0.405	0.647
Planktic foram div.	0.256	0.206	-1.025	1.73	0.442	-0.904
Sea level	0.251	-0.078	-1.416	1.139	0.531	2.839
Sea surface temp.	0.445	-0.877	-2.616	0.457	0.432	-1.648
Exponential (s)						
Diversity dep.	0.891	-5.61	-15.448	-0.51	0.746	-2.76
Ichthyolith abund.	0.688	0.868	-6.305	8.836	0.64	0.795
Nannoplankton div.	0.649	1.626	-2.182	7.62	0.613	0.07
pCO_2	0.79	-4.747	-17.709	1.526	0.924	-9.89
Planktic foram div.	0.578	1.151	-2.001	6.251	0.642	1.634
Sea level	0.872	9.999	-2.13	34.017	0.973	17.978
Sea surface temp.	0.746	0.218	-9.584	12.231	0.687	-2.24
Linear (s)						
Diversity dep.	0.888	-5.466	-15.07	-0.381	0.74	-2.987
Ichthyolith abund.	0.678	0.386	-7.344	7.662	0.633	0.552
Nannoplankton div.	0.647	1.757	-2.22	8.431	0.605	0.043
pCO_2	0.787	-5.307	-19.325	1.655	0.924	-9.383
Planktic foram div.	0.574	1.329	-1.945	7.222	0.634	1.918
Sea level	0.864	11.14	-1.583	38.886	0.971	17.913
Sea surface temp.	0.731	-0.42	-9.796	9.615	0.677	-2.601

g = genus; s = species; ex = extinction; sp = speciation; W = correlation shrinkage weight

Supplementary Table 11. Late Cretaceous W. Int. Seaway ammonoid diversification drivers

	W (sp)	l95 (sp)	u95 (sp)	W (ex)	l95 (ex)	u95 (ex)
Exponential (g)						
Diversity dep.	0.182	0.015	-0.732	0.878	0.358	0.705
Ichthyolith abund.	0.206	0.225	-0.697	1.52	0.561	-1.123
Nannoplankton div.	0.195	-0.014	-0.733	0.733	0.338	0.044
pCO_2	0.263	-0.349	-1.049	0.215	0.287	0.336
Planktic foram div.	0.236	0.192	-0.82	1.717	0.212	0.088
Sea level	0.272	-0.31	-1.171	0.412	0.312	0.548
Sea surface temp.	0.249	-0.292	-0.907	0.321	0.431	-1.01
Linear (g)						
Diversity dep.	0.184	0.03	-0.744	0.928	0.36	0.699
Ichthyolith abund.	0.209	0.222	-0.727	1.491	0.566	-1.109
Nannoplankton div.	0.201	-0.021	-0.714	0.768	0.338	0.028
pCO_2	0.265	-0.354	-1.064	0.207	0.29	0.327
Planktic foram div.	0.245	0.203	-0.821	1.88	0.213	0.087
Sea level	0.271	-0.303	-1.145	0.421	0.317	0.544
Sea surface temp.	0.252	-0.296	-0.923	0.312	0.432	-1.011
Exponential (s)						
Diversity dep.	0.339	0.399	-0.08	0.941	0.574	1.24
Ichthyolith abund.	0.36	-0.332	-1.494	0.601	0.561	-1.251
Nannoplankton div.	0.302	0.23	-0.546	1.527	0.413	0.542
pCO_2	0.522	-0.873	-1.524	-0.346	0.59	-1.267
Planktic foram div.	0.411	-0.526	-1.052	0.181	0.297	0.29
Sea level	0.326	-0.308	-1.424	0.541	0.704	2.058
Sea surface temp.	0.281	0.119	-0.631	1.086	0.628	-1.341
Linear (s)						
Diversity dep.	0.336	0.391	-0.091	0.916	0.575	1.261
Ichthyolith abund.	0.356	-0.362	-1.703	0.629	0.562	-1.23
Nannoplankton div.	0.304	0.276	-0.538	1.93	0.418	0.546
pCO_2	0.525	-0.889	-1.652	-0.358	0.59	-1.251
Planktic foram div.	0.413	-0.521	-1.045	0.201	0.299	0.306
Sea level	0.326	-0.304	-1.295	0.577	0.704	2.04
Sea surface temp.	0.288	0.137	-0.632	1.158	0.632	-1.309

g = genus; s = species; ex = extinction; sp = speciation; W = correlation shrinkage weight

Supplementary Table 12. Late Cretaceous West Pacific ammonoid diversification drivers

	W (sp)	l95 (sp)	u95 (sp)	W (ex)	l95 (ex)	u95 (ex)
Exponential (g)						
Diversity dep.	0.463	-0.872	-2.845	0.472	0.404	-0.363
Ichthyolith abund.	0.405	0.703	-0.526	2.595	0.406	-0.388
Nannoplankton div.	0.311	-0.084	-1.368	1.205	0.755	4.312
pCO_2	0.538	1.669	-0.471	5.813	0.442	0.765
Planktic foram div.	0.407	-0.436	-2.797	1.325	0.547	-1.594
Sea level	0.385	-0.586	-3.217	1.038	0.343	0.041
Sea surface temp.	0.488	-0.981	-3.09	0.553	0.398	-0.534
Linear (g)						
Diversity dep.	0.46	-0.827	-2.553	0.478	0.403	-0.358
Ichthyolith abund.	0.402	0.704	-0.526	2.692	0.404	-0.418
Nannoplankton div.	0.307	-0.104	-1.424	1.13	0.75	4.027
pCO_2	0.536	1.594	-0.454	5.332	0.441	0.744
Planktic foram div.	0.407	-0.459	-3	1.34	0.544	-1.519
Sea level	0.378	-0.562	-2.913	1.031	0.34	0.038
Sea surface temp.	0.491	-0.97	-3.099	0.593	0.397	-0.532
Exponential (s)						
Diversity dep.	0.506	-1.931	-7.03	0.349	0.255	0.098
Ichthyolith abund.	0.258	-0.249	-2.862	1.536	0.277	0.193
Nannoplankton div.	0.343	1.291	-0.814	7.236	0.354	2.323
pCO_2	0.307	0.707	-1.14	4.495	0.269	0.011
Planktic foram div.	0.295	0.754	-1.078	4.561	0.307	0.746
Sea level	0.27	0.27	-1.985	2.929	0.324	0.788
Sea surface temp.	0.296	0.341	-1.959	3.62	0.29	0.354
Linear (s)						
Diversity dep.	0.476	-2.061	-7.628	1.147	0.607	-1.409
Ichthyolith abund.	0.222	1.838	-2.799	9.916	0.49	0.105
Nannoplankton div.	0.305	-2.048	-9.811	1.995	0.557	-0.058
pCO_2	0.266	0.3	-4.403	5.878	0.619	-2.501
Planktic foram div.	0.252	1.859	-3.21	10.444	0.506	0.953
Sea level	0.238	3.58	-1.698	13.994	0.896	10.932
Sea surface temp.	0.248	0.949	-7.599	10.506	0.656	-3.085

g = genus; s = species; ex = extinction; sp = speciation; W = correlation shrinkage weight

Supplementary Table 13. Suborder predictors of regional and global Late Cretaceous ammonoid extinction risk

	I	Ancyloceratina	Haploceratina	Lytoceratina	Perisphinctina	Phylloceratina
Antarctic (g)	0.043142	0.172282	0.20603	0.203906	0.206226	0.211556
Antarctic (s)	0.20586	0.173989	0.202792	0.219516	0.179142	0.224561
E. Pacific (g)	0.667207	0.154449	0.232061	0.113681	0.361645	0.138164
E. Pacific (s)	0.963716	0.20665	0.212239	0.048004	0.306492	0.226615
Gulf (g)	1	0.057449	0.837121	0.004529	0.095476	0.005425
Gulf (s)	0.259352	0.137955	0.265498	0.192133	0.229489	0.174925
Global (g)	0.945511	0.178933	0.228324	0.115769	0.393984	0.08299
Global (s)	0.942519	0.202714	0.178402	0.129786	0.385759	0.103339
S. Africa (g)	0.727057	0.115595	0.143564	0.281928	0.359256	0.099657
S. Africa (s)	0.52394	0.136902	0.172021	0.267416	0.279991	0.159633
Tethys (g)	0.880175	0.209309	0.211643	0.069961	0.440341	0.068747
Tethys (s)	0.999002	0.153479	0.547591	0.043128	0.213959	0.041843
W. Africa (g)	0.428928	0.158667	0.231317	0.17002	0.271297	0.168699
W. Africa (s)	0.025187	0.326459	-	0.339322	0.334219	-
WIS (g)	0.329551	0.199608	0.220902	0.17153	0.238722	0.169239
WIS (s)	0.657481	0.180419	0.200834	0.203498	0.259876	0.155373
W. Pacific (g)	0.425561	0.180517	0.207002	0.183271	0.333148	0.282364
W. Pacific (s)	0.03192	0.363465	0.264975	0.333014	0.376928	-

Supplementary Table 14. Superfamily predictors of regional and global Late Cretaceous ammonoid extinction risk

	I	Acanthoceratoidea	Ancyloceratoidea	Deshayesitoidea	Desmoceratoidea	Douvilleiceratoidea	Haploceratoidea
Antarctic (g)	0.041022	0.110131	-	-	0.12725	-	0.129212
Antarctic (s)	0.301372	0.086793	-	-	0.177263	-	0.115059
E. Pacific (g)	0.411222	0.211973	0.1263	-	0.106172	-	0.140916
E. Pacific (s)	0.171446	0.092002	-	-	0.119196	-	0.136421
Gulf (g)	0.996758	0.105701	0.449986	-	0.019664	0.070284	0.177127
Gulf (s)	0.993267	0.226888	-	-	0.066238	0.071463	0.221781
Global (g)	0.20586	0.068651	0.118056	0.08389	0.075943	0.085426	0.080211
Global (s)	0.933042	0.115628	-	0.067118	0.046436	0.099676	0.088121
S. Africa (g)	0.07818	0.093483	-	0.115427	0.126243	-	0.127993
S. Africa (s)	0.1	0.099615	-	-	0.127149	-	0.128475
Tethys (g)	0.724065	0.313904	-	0.127849	0.059506	0.136366	0.091277
Tethys (s)	0.878928	0.150298	-	0.226304	0.046808	0.195933	0.211555
W. Africa (g)	0.05399	0.103429	-	-	0.132301	-	0.133437
W. Africa (s)	0.048254	0.145432	-	-	0.174926	-	-
WIS (g)	0.080673	0.085996	-	0.097902	0.105516	0.106259	0.108528
WIS (s)	0.151247	0.099457	-	-	0.104114	0.105989	0.109283
W. Pacific (g)	0.294514	0.218345	-	-	0.133195	-	0.149753
W. Pacific (s)	0.115586	0.183232	-	-	0.175568	-	0.154815

Supplementary Table 14 (continued). Superfamily predictors of regional and global Late Cretaceous ammonoid extinction risk

	I	Hoplitoidea	Lytoceratoidea	Phylloceratoidea	Scaphitoidea	Tetragonitoidea	Turrilitoidea
Antarctic (g)	0.041022	0.110131	-	-	0.12725	-	0.129212
Antarctic (s)	0.301372	0.086793	-	-	0.177263	-	0.115059
E. Pacific (g)	0.411222	0.211973	0.1263	-	0.106172	-	0.140916
E. Pacific (s)	0.171446	0.092002	-	-	0.119196	-	0.136421
Gulf (g)	0.996758	0.105701	0.449986	-	0.019664	0.070284	0.177127
Gulf (s)	0.993267	0.226888	-	-	0.066238	0.071463	0.221781
Global (g)	0.20586	0.068651	0.118056	0.08389	0.075943	0.085426	0.080211
Global (s)	0.933042	0.115628	-	0.067118	0.046436	0.099676	0.088121
S. Africa (g)	0.07818	0.093483	-	0.115427	0.126243	-	0.127993
S. Africa (s)	0.1	0.099615	-	-	0.127149	-	0.128475
Tethys (g)	0.724065	0.313904	-	0.127849	0.059506	0.136366	0.091277
Tethys (s)	0.878928	0.150298	-	0.226304	0.046808	0.195933	0.211555
W. Africa (g)	0.05399	0.103429	-	-	0.132301	-	0.133437
W. Africa (s)	0.048254	0.145432	-	-	0.174926	-	-
WIS (g)	0.080673	0.085996	-	0.097902	0.105516	0.106259	0.108528
WIS (s)	0.151247	0.099457	-	-	0.104114	0.105989	0.109283
W. Pacific (g)	0.294514	0.218345	-	-	0.133195	-	0.149753
W. Pacific (s)	0.115586	0.183232	-	-	0.175568	-	0.154815
