# Supplementary material for: Late Cretaceous ammonoids show that drivers of diversification are regionally heterogeneous
Source: Nat Commun. 2024 Jun 27;15:5382. doi: 10.1038/s41467-024-49462-z (PMC11211348; doi:10.1038/s41467-024-49462-z)
Supplement: Supplementary file 3 — Description of Additional Supplementary Files [file 41467_2024_49462_MOESM3_ESM.pdf]

Description of Additional Supplementary Files

File Name: Supplementary Data 1
Description: Correlations between Late Cretaceous ammonoid spatial extent and subsampled diversity

File Name: Supplementary Data 2
Description: Fits of PyRate preservation models to spatially standardised regional and global Late Cretaceous ammonoid occurrence datasets

File Name: Supplementary Data 3
Description: Fits of exponential and linear birth-death correlation models to regional and global Late Cretaceous ammonoid diversification rates

File Name: Supplementary Data 4
Description: Late Cretaceous Antarctic ammonoid diversification drivers

File Name: Supplementary Data 5
Description: Late Cretaceous East Pacific ammonoid diversification drivers

File Name: Supplementary Data 6
Description: Late Cretaceous Atlantic and Gulf ammonoid diversification drivers

File Name: Supplementary Data 7
Description: Late Cretaceous global ammonoid diversification drivers

File Name: Supplementary Data 8
Description: Late Cretaceous South African ammonoid diversification drivers

File Name: Supplementary Data 9
Description: Late Cretaceous Tethyan ammonoid diversification drivers

File Name: Supplementary Data 10
Description: Late Cretaceous West African ammonoid diversification drivers

File Name: Supplementary Data 11
Description: Late Cretaceous W. Int. Seaway ammonoid diversification drivers

File Name: Supplementary Data 12
Description: Late Cretaceous West Pacific ammonoid diversification drivers

File Name: Supplementary Data 13
Description: Suborder predictors of regional and global Late Cretaceous ammonoid extinction risk

File Name: Supplementary Data 14
Description: Superfamily predictors of regional and global Late Cretaceous ammonoid extinction risk
