# Supplementary material for: Late Cretaceous ammonoids show that drivers of diversification are regionally heterogeneous
Source: Nat Commun. 2024 Jun 27;15:5382. doi: 10.1038/s41467-024-49462-z (PMC11211348; doi:10.1038/s41467-024-49462-z)
Supplement: Supplementary file 18 — Reporting Summary [file 41467_2024_49462_MOESM18_ESM.pdf]

nature portfolio

Corresponding author(s): Joseph Flannery Sutherland

Last updated by author(s): May 9, 2024

Reporting Summary

Nature Portfolio wishes to improve the reproducibility of the work that we publish. This form provides structure for consistency and transparency in reporting. For further information on Nature Portfolio policies, see our Editorial Policies and the Editorial Policy Checklist.

Statistics

For all statistical analyses, confirm that the following items are present in the figure legend, table legend, main text, or Methods section.

n/a	Confirmed	
☐	☒	The exact sample size (*n*) for each experimental group/condition, given as a discrete number and unit of measurement
☐	☒	A statement on whether measurements were taken from distinct samples or whether the same sample was measured repeatedly
☐	☒	The statistical test(s) used AND whether they are one- or two-sided<br>*Only common tests should be described solely by name; describe more complex techniques in the Methods section.*
☐	☒	A description of all covariates tested
☒	☐	A description of any assumptions or corrections, such as tests of normality and adjustment for multiple comparisons
☒	☐	A full description of the statistical parameters including central tendency (e.g. means) or other basic estimates (e.g. regression coefficient) AND variation (e.g. standard deviation) or associated estimates of uncertainty (e.g. confidence intervals)
☒	☐	For null hypothesis testing, the test statistic (e.g. *F*, *t*, *r*) with confidence intervals, effect sizes, degrees of freedom and *P* value noted<br>*Give P values as exact values whenever suitable.*
☐	☒	For Bayesian analysis, information on the choice of priors and Markov chain Monte Carlo settings
☐	☒	For hierarchical and complex designs, identification of the appropriate level for tests and full reporting of outcomes
☐	☒	Estimates of effect sizes (e.g. Cohen's *d*, Pearson's *r*), indicating how they were calculated

Our web collection on statistics for biologists contains articles on many of the points above.

Software and code

Policy information about availability of computer code

Data collection	Data compilation was performed by several of the authors. R was used to download publically available ammonoid occurrence data from the Paleobiology Database. The other raw datasets were compiled manually from the primary literature or direct observation of museum specimens as excel spreadsheets. Database compilation from these sources was performed in R
Data analysis	Software: R (v 4.2.2), PyRate (v 3.0), Tracer (v 1.7.2). R packages: fossilbrush (no version), icosa (v 0.11.0), iNEXT (v 3.0.0) .All software is open source and freely available to download. All code and data is publically available on figshare at: https://doi.org/10.6084/m9.figshare.25563633

For manuscripts utilizing custom algorithms or software that are central to the research but not yet described in published literature, software must be made available to editors and reviewers. We strongly encourage code deposition in a community repository (e.g. GitHub). See the Nature Portfolio guidelines for submitting code & software for further information.

Data

Policy information about availability of data

All manuscripts must include a data availability statement. This statement should provide the following information, where applicable:

- Accession codes, unique identifiers, or web links for publicly available datasets
- A description of any restrictions on data availability
- For clinical datasets or third party data, please ensure that the statement adheres to our policy

All data (raw occurrence data files, compiled data sets, environmental time series and palaeogeographic maps) are available in the electronic supplement. The supplement link will be added to the Data Availability statement if accepted for publication

Research involving human participants, their data, or biological material

Policy information about studies with human participants or human data. See also policy information about sex, gender (identity/presentation), and sexual orientation and race, ethnicity and racism.

Reporting on sex and gender	NA
Reporting on race, ethnicity, or other socially relevant groupings	NA
Population characteristics	NA
Recruitment	NA
Ethics oversight	NA

Note that full information on the approval of the study protocol must also be provided in the manuscript.

Field-specific reporting

Please select the one below that is the best fit for your research. If you are not sure, read the appropriate sections before making your selection.

☐ Life sciences ☐ Behavioural & social sciences ☒ Ecological, evolutionary & environmental sciences

For a reference copy of the document with all sections, see nature.com/documents/nr-reporting-summary-flat.pdf

Ecological, evolutionary & environmental sciences study design

All studies must disclose on these points even when the disclosure is negative.

Study description	Diversification rates and drivers inferred for regional and global occurrence datasets of Late Cretaceous ammonoids
Research sample	19,536 mostly substage-level Late Cretaceous ammonoid fossil occurrences (mean and median age uncertainties of 3.5 and 2.5 Ma, respectively) classified at the order, suborder, superfamily, and genus levels, with additional species classifications for most.
Sampling strategy	No sample sizes were predetermined. Samples were determined by inclusion of occurrences within bioregions selected to reflect the spatial sampling availability of the ammonoid fossil record, along with patterns of endemism where possible
Data collection	Downloads from the Paleobiology Database, compilation from the primary literature, compilation from observed museum specimens
Timing and spatial scale	Paleobiology Database data was downloaded on 31/05/22. Other datasets were compiled at different times within approximately 6 years of the submission date (such compilations are slow to create, so their times of completion are generally much closer to the submission date)
Data exclusions	All data exclusions were for occurrences with suspect taxonomy or stratigraphy. These rejections are documented in the manuscript and are explicitly listed in the electronic supplement
Reproducibility	The data used for all analyses is static within the electronic supplement and can be re-analysed using the exact R and bash scripts provided as well. The Bayesian analyses are not guaranteed to retain exactly the same numeric values by their nature, but the results will be unaffected by these negligible discrepancies
Randomization	Allocation was not random, but based on geographic location. This is crucial as we are examining regional variation in diversification. The uncertain stratigraphic ages of the occurrences were randomised 10 times within their individual ranges (described in manuscript) then the final results averaged across these replicates
Blinding	Blinding was not relevant

Did the study involve field work? ☐ Yes ☒ No

Reporting for specific materials, systems and methods

We require information from authors about some types of materials, experimental systems and methods used in many studies. Here, indicate whether each material, system or method listed is relevant to your study. If you are not sure if a list item applies to your research, read the appropriate section before selecting a response.

Materials & experimental systems

n/a	Involved in the study
☒	☐ Antibodies
☒	☐ Eukaryotic cell lines
☐	☒ Palaeontology and archaeology
☒	☐ Animals and other organisms
☒	☐ Clinical data
☒	☐ Dual use research of concern
☒	☐ Plants

Methods

n/a	Involved in the study
☒	☐ ChIP-seq
☒	☐ Flow cytometry
☒	☐ MRI-based neuroimaging

Palaeontology and Archaeology

Specimen provenance	Global Late Cretaceous ammonoid occurrence records from the Paleobiology Database, primary literature and museum collections
Specimen deposition	No new specimens were collected for this study, so no deposition was required
Dating methods	No new dates were provided

☒ Tick this box to confirm that the raw and calibrated dates are available in the paper or in Supplementary Information.

Ethics oversight	No ethical guidance was needed

Note that full information on the approval of the study protocol must also be provided in the manuscript.

Plants

Seed stocks	NA
Novel plant genotypes	NA
Authentication	NA
